# 11β-Hydroxysteroid Dehydrogenase Type 1 Gene Knockout Attenuates Atherosclerosis and In Vivo Foam Cell Formation in Hyperlipidemic apoE^−/−^ Mice

**DOI:** 10.1371/journal.pone.0053192

**Published:** 2013-02-01

**Authors:** Ricardo A. García, Debra J. Search, John A. Lupisella, Jacek Ostrowski, Bo Guan, Jian Chen, Wen-Pin Yang, Amy Truong, Aiqing He, Rongan Zhang, Mujing Yan, Samuel E. Hellings, Peter S. Gargalovic, Carol S. Ryan, Linda M. Watson, Robert A. Langish, Petia A. Shipkova, Nancy L. Carson, Joseph R. Taylor, Richard Yang, George C. Psaltis, Thomas W. Harrity, Jeffrey A. Robl, David A. Gordon

**Affiliations:** 1 Cardiovascular Drug Discovery, Bristol-Myers Squibb Company, Pennington, New Jersey, United States of America; 2 Applied Genomics, Bristol-Myers Squibb Company, Pennington, New Jersey, United States of America; 3 Pharmaceutical Compound Optimization: Discovery Toxicology, Bristol-Myers Squibb Company, Lawrenceville, New Jersey, United States of America; 4 Pharmaceutical Compound Optimization: Discovery Analytical Sciences, Bristol-Myers Squibb Company, Pennington, New Jersey, United States of America; 5 Metabolic Diseases, Bristol-Myers Squibb Company, Pennington, New Jersey, United States of America; 6 Veterinary Sciences, Bristol-Myers Squibb Company, Pennington, New Jersey, United States of America; 7 Discovery Chemistry, Bristol-Myers Squibb Company, Pennington, New Jersey, United States of America; Fudan University, China

## Abstract

**Background:**

Chronic glucocorticoid excess has been linked to increased atherosclerosis and general cardiovascular risk in humans. The enzyme 11β-hydroxysteroid dehydrogenase type 1 (11βHSD1) increases active glucocorticoid levels within tissues by catalyzing the conversion of cortisone to cortisol. Pharmacological inhibition of 11βHSD1 has been shown to reduce atherosclerosis in murine models. However, the cellular and molecular details for this effect have not been elucidated.

**Methodology/Principal Findings:**

To examine the role of 11βHSD1 in atherogenesis, 11βHSD1 knockout mice were created on the pro-atherogenic apoE^−/−^ background. Following 14 weeks of Western diet, aortic cholesterol levels were reduced 50% in 11βHSD1^−/−^/apoE^−/−^ mice vs. 11βHSD1^+/+^/apoE^−/−^ mice without changes in plasma cholesterol. Aortic 7-ketocholesterol content was reduced 40% in 11βHSD1^−/−^/apoE^−/−^ mice vs. control. In the aortic root, plaque size, necrotic core area and macrophage content were reduced ∼30% in 11βHSD1^−/−^/apoE^−/−^ mice. Bone marrow transplantation from 11βHSD1^−/−^/apoE^−/−^ mice into apoE^−/−^ recipients reduced plaque area 39–46% in the thoracic aorta. In vivo foam cell formation was evaluated in thioglycollate-elicited peritoneal macrophages from 11βHSD1^+/+^/apoE^−/−^ and 11βHSD1^−/−^/apoE^−/−^ mice fed a Western diet for ∼5 weeks. Foam cell cholesterol levels were reduced 48% in 11βHSD1^−/−^/apoE^−/−^ mice vs. control. Microarray profiling of peritoneal macrophages revealed differential expression of genes involved in inflammation, stress response and energy metabolism. Several toll-like receptors (TLRs) were downregulated in 11βHSD1^−/−^/apoE^−/−^ mice including TLR 1, 3 and 4. Cytokine release from 11βHSD1^−/−^/apoE^−/−^-derived peritoneal foam cells was attenuated following challenge with oxidized LDL.

**Conclusions:**

These findings suggest that 11βHSD1 inhibition may have the potential to limit plaque development at the vessel wall and regulate foam cell formation independent of changes in plasma lipids. The diminished cytokine response to oxidized LDL stimulation is consistent with the reduction in TLR expression and suggests involvement of 11βHSD1 in modulating binding of pro-atherogenic TLR ligands.

## Introduction

Glucocorticoids are ubiquitous mammalian hormones involved in the regulation of several fundamental biological processes including energy metabolism, inflammation, arousal, cognition and the response to physiological stress. In humans, the primary active glucocorticoid hormone, cortisol, binds to intracellular glucocorticoid and mineralocorticoid receptors found in target tissues. Dysfunctional regulation of glucocorticoid metabolism resulting in excess cortisol in tissues such as adipose, liver and the vasculature has been implicated as a key mediator in the pathogenesis of obesity, type 2 diabetes and cardiovascular disease [1]–[3]. Growing epidemiological evidence suggests that glucocorticoid excess may also contribute to the progression of atherosclerosis [3]. Although not completely characterized, the evidence suggests that direct interactions of glucocorticoids with cells in the vasculature may potentiate plaque development [4] [3] [5] [6] independent of changes in plasma risk factors such as cholesterol.

Endogenous cortisol levels (corticosterone in mice) are regulated by two distinct pathways. The hypothalamic-pituitary-adrenal axis is the pathway classically associated with regulation of plasma cortisol. In addition, cortisol tone is also regulated intracellularly by the enzymatic activities of two isoforms of 11β-hydroxysteroid dehydrogenase, type 1 (“11βHSD1”) and type 2 (“11βHSD2”). The latter, 11βHSD2, is mainly expressed in aldosterone-target tissues such as kidney, colon and salivary glands [7]. 11βHSD2 converts active cortisol/corticosterone to the inactive form cortisone/11-dehydrocorticosterone, thereby limiting ligand availability for mineralocorticoid receptor binding in these tissues. By contrast, 11βHSD1 is expressed in tissues with high sensitivity to glucocorticoids such as liver, adipose, brain and lung [7]. 11βHSD1 converts inactive substrate to the active hormone thereby stimulating glucocorticoid and mineralocorticoid receptor activation. In addition, 11βHSD1 has also been shown to possess oxysterol metabolizing properties via its keto-reductase activity [8]–[9]. Although the pro-atherogenic properties of oxysterols such as 7-ketocholesterol have been described, [10]–[11] the overall impact of this enzymatic activity on disease development is unclear.

Glucocorticoid amplification by increased 11βHSD1 activity in highly metabolic tissues such as adipose and liver is proposed to potentiate a phenotype resembling the metabolic syndrome [2] [7]. This concept has been rigorously tested in preclinical models of disease. In the Zucker fatty rat, increased 11βHSD1 activity was shown to positively correlate with plasma corticosterone levels and omental fat mass [12]. In the mouse, selective overexpression of 11βHSD1 in adipose tissue yields a distinctive phenotype of visceral obesity with many features of the metabolic syndrome [13]. Conversely, 11βHSD1 knockout mice do not develop characteristics of the metabolic syndrome when placed on a high-fat diet. Fat-fed 11βHSD1 knockout mice have reduced visceral fat accumulation, increased insulin sensitization, enhanced glucose tolerance and improvements in lipid and lipoprotein profiles [14]–[16]. 11βHSD1 inhibitor studies in the mouse have shown that selective pharmacological inhibition can positively impact metabolic endpoints in the setting of diet-induced obesity [17] [7]. These observations have stimulated significant efforts towards development of an inhibitor to treat type 2 diabetes mellitus.

The beneficial effects of 11βHSD1 inhibition on atherosclerosis have been demonstrated in the mouse with small-molecule 11βHSD1 inhibitors [17] [18]. In these studies, plaque reductions were observed in conjunction with improvements in metabolic endpoints and overall lowering of pro-atherogenic lipids. Thus, it remains unclear whether atheroprotection via 11βHSD1 inhibition is attributed to improvements in glucose/insulin handling, lipid lowering, a direct effect of 11βHSD1 inhibition at the vessel wall or some combination of the above. In addition, the molecular pathways that mediate the atheroprotective effect of 11βHSD1 have not been elucidated.

To clarify these issues, 11βHSD1-deficient mice were created on the apoE knockout background to investigate the propensity of atherosclerosis development. The impact of 11βHSD1 deficiency on plasma lipid profiles, vessel wall atherosclerosis and macrophage function was evaluated in Western diet-fed mice. Intrinsic differences in gene expression profiles and biological pathways affected by 11βHSD1 gene deficiency were evaluated by microarray-based gene profiling of thioglycollate-elicited macrophages from hyperlipidemic mice. The results of these analyses and their implications are described herein.

## Methods

### Animals and Diet

Animal studies were performed according to guidelines established by the American Association for Accreditation of Laboratory Animal Care and protocols were approved by the Bristol-Myers Squibb-Hopewell Animal Care and Use Committee. 11βHSD1 knockout mice were created at Lexicon Pharmaceuticals (The Woodlands, TX) as described in Figure S1. 11βHSD1 knockout mice were backcrossed onto a C57BL/6 background for more than 10 generations and mated with apoE knockout mice on the C57BL/6 background obtained from The Jackson Laboratory (Bar Harbor, ME) to create litters on an apoE-deficient background. Animal breeding was carried out at Bristol-Myers Squibb under pathogen-free barrier conditions. Genotypes of litters were characterized by isolated genomic DNA analysis and grouped according to sex and genotype. All mice were fed standard rodent chow during the acclimation phase. At ∼8 weeks of age, mice were fed Western Diet D12079B (Research Diets, New Brunswick, NJ) containing 0.2% cholesterol and 20% milk fat for 5 weeks (peritonitis studies) or 14 weeks (diet-induced atherosclerosis studies). Mice were euthanized under isoflurane anesthesia followed by inhalation of CO_2_ for collection of blood, hearts, aortas and archival tissues. All in vivo studies were repeated at least in duplicate to assess reproducibility and to increase statistical power.

### Bone Marrow Transplantation

Male apoE^−/−^ mice (8–10 weeks of age; B6.129P2-Apoe^tm1Unc^/J) purchased from The Jackson Laboratory were subjected to whole body irradiation to induce bone marrow aplasia using a Mark I Model 30 irradiator (JL Shepherd & Associates, San Francisco, CA). Irradiation was carried out with a single exposure of 1000 rads using a cesium radiation source. Donor bone marrow cells were harvested from age-matched male 11β^−/−^/apoE^−/−^ and 11β^+/+^/apoE^−/−^ littermate control mice by flushing isolated femurs and tibias with RPMI supplemented with 20 mM HEPES, 10 U/ml heparin and 100 U/ml penicillin/streptomycin. Approximately four hours after irradiation, mice were anesthetized with isoflurane (2–4%) and immediately reconstituted with 5–7 million bone marrow cells given intravenously (∼200 µl) via the retro-orbital sinus. Following bone marrow transplantation, mice were housed in autoclaved cages supplied with HEPA-filtered air and fed regular chow for 4 weeks to enable bone marrow reconstitution. A small group of irradiated control mice that were injected with cell-free vehicle did not survive beyond two weeks post irradiation. Bone marrow transplant-recipient mice were switched to Western diet (Research Diets, D12079B) for 12 weeks to stimulate atherogenesis.

Confirmation of bone marrow reconstitution was carreid out by RT-PCR analysis of whole blood leukocytes, as described (*vide infra*). Atherosclerosis development was analyzed by en face evaluation of excised aortas using oil red O.

### Blood Pressure Measurements

Blood pressures (systolic, diastolic and mean arterial pressure) were measured in conscious hyperlipidemic 11β^−/−^/apoE^−/−^ and 11β^+/+^/apoE^−/−^ littermate control mice using a non-invasive computerized tail cuff system (CODA Non-Invasive Blood Pressure Monitor, Kent Scientific Corporation, Torrington, CT). Mice were conditioned to tail cuff instrumentation over several days to control for stress. As a normolipidemic reference, blood pressures were also measured in chow-fed male C57BL/6 mice. Blood pressure analyses consisted of fifteen tail cuff pressure acquisitions per run. Data for individual animals represent the average of at least five high-quality acquisitions.

### Plasma Lipids Analysis

EDTA-anti-coagulated blood samples were taken by retro-orbital bleeding following a 4 hr fast and plasma was isolated by centrifugation. Plasma total cholesterol, triglycerides, HDL-cholesterol and non-HDL-cholesterol were analyzed enzymatically using a Siemens Advia 1800 automated chemistry analyzer (Siemens Healthcare Diagnostics, Flanders NJ).

### Whole Blood RNA Isolation

Whole blood was diluted in 1X (final) Nucleic Acid Purification Lysis Buffer (#4305895, Applied Biosystems Inc., Foster City, CA). Total RNA was isolated using an Applied Biosystems Prism 6100 Nucleic Acid Prep Station according to the manufacturer's instructions.

### Atherosclerosis Analysis

The degree of atherosclerosis development was assessed by aortic lipid extraction (diet-induced atherosclerosis studies) or en face analysis using oil red O stain (bone marrow transplantation studies). Following euthanasia, hearts and aortas were perfused with heparinzed saline solution (0.9% NaCl) followed by 10% formalin. Hearts were stored in formalin. Thoracic aortae were harvested from mice, adventitial connective tissue removed and aortae were delipidated with ethyl acetate:acetone (2∶1 vol/vol) supplemented with 0.01% 2,6,-di-tert-butyl-4-methylphenol. Samples were gently agitated overnight at 37°C and blown dry under nitrogen. Aortae were dried and delipidated dry weights were measured. The dried lipid film from each aorta was resuspended in 0.3 ml of 10% Triton X-100 with gentle agitation at 37°C for 90 minutes. Samples were analyzed by enzymatic assay for total cholesterol using a Roche Cobas Mira Chemistry Analyzer. Cholesterol content was reported with respect to aorta dry weight.

En face staining of the isolated thoracic aorta was carried out using oil red O. The aortic arch and descending aorta were opened longitudinally and pinned to a black wax plate with Austrian fine stainless steel pins (Fine Science Tools, #26002-15). Each aorta was rinsed with 70% ethanol, stained with oil red O and de-stained in 70% ethanol. Following a brief rinse with de-ionized water, pinned aortae were immersed in phosphate-buffered saline and photographed using a Nikon Digital Camera (Nikon Digital Sight DS-Fi1) mounted on Nikon Dissecting Microscope (SMZ 1000, Micron-Optics, Cedar Knolls, NJ). Image analysis was performed using NIS-Elements: Basic Research Version 3.0 software (Micron-Optics, Cedar Knolls, NJ). Lesion area was expressed as a percentage of the total measured aortic area.

To characterize plaque composition, histological analysis of lesions in the aortic root was performed via the Paigen method [19]. Serial sections were stained with trichrome for lesion area measurements. Plaque necrosis area was analyzed from trichrome-stained aortic root images based on acellularity within the intima and/or presence of crystalline clefts [20]. Macrophage content was evaluated by CD68-positive staining via immunohistochemistry. A rat anti-mouse CD68 antibody (Cat. #: MCA1957; AbD Serotec, Raleigh, NC) was applied to paraffin-embedded and sectioned samples. The antigen:antibody binding complex was detected using an anti-rat polymer system directly conjugated to horse radish peroxidase (BioCare Medical, Concord, CA). Sections were counterstained with Hematoxylin, Gills Formula (Vector Labs, Burlingame, CA), dehydrated and cleared with xylene. The antibody required heat induced epitope retrieval for one minute using a citrate-based retrieval solution at pH 6.0 (Biocare Medical, Concord, CA) prior to the start of the immunolabeling procedure. Isotyped-matched controls were simultaneously run for each sample.

### Aortic Oxysterol Analysis

Samples were reconstituted in 150 µL of methanol and briefly vortexed. A volume of 130 µL was transferred to 96 well plates for LCMS analysis. A Waters Acquity UPLC System (Milford, MA) in line with a Thermo Fisher LTQ Orbitrap mass spectrometer (Waltham, MA) was used to separate and detect components with mass accuracy <5 ppm. The mass spectrometer was operated in full scan, positive electrospray (+ESI) mode. Reverse-phase gradient LC conditions were employed using a Water Acquity BEH C18 2.1×100 mm 1.7 mm column and acetonitrile-water mobile phases. Component peak integration was performed using Thermo-Fisher Xcalibur Quan Browser software. An accurate mass window of 10 ppm was used to process the following components: cholesterol  = 369.3504 (ms source water loss), 7β-hydroxycholesterol  = 385.3494 (ms source water loss) 7-ketocholesterol  = 401.3405; D7-7β-hydroxycholesterol (internal standard)  = 392.3898 (ms source water loss).

### In Vivo Foam Cell Studies

In vivo foam cell studies were performed with mice starting at 8 weeks of age and fed a Western diet for 4 weeks (Western diet, 12079B, 20% fat 0.2% cholesterol, Research Diets). Following the dietary lead-in phase, mice were injected with 1.5 ml of a 4% thioglycollate solution to induce peritonitis. Four days post-injection, mice were euthanized and the abdominal cavity was lavaged with 10 ml of ice-cold phosphate-buffered saline to collect peritoneal macrophages. Approximately 100 µl of each cell suspension was analyzed for total cell count via automated cell counting and cell type using modified Wright-Giemsa staining and light microscope evaluation. For analysis of lipid droplet accumulation, cells were stained using oil red O/hematoxylin [21]. Remaining cell suspensions were transferred to pre-weighed glass vials and centrifuged at 1200 rpm for 4 minutes. Supernatant devoid of cells was removed and each cell pellet was mixed with ethyl acetate:acetone (2∶1 vol/vol) supplemented with 0.01% 2,6,-di-tert-butyl-4-methylphenol overnight at 37°. Samples were blown dry under nitrogen and resuspended in 100 µl of 10% Triton X-100 with agitation at 37°C for 90 minutes. Samples were analyzed for total cholesterol content by enzymatic analysis using a Roche Cobas Mira Chemistry Analyzer.

### Cytokine Studies

Thioglycollate-elicited peritoneal macrophage/foam cells were prepared as described above. Macrophages were plated in DMEM (1 million cells/ml) supplemented with 20% serum and 200 nM 11-dehydrocorticosterone at 37°C for 5 hours. Adherent cells were washed twice with phosphate-buffered saline and incubated with serum-free DMEM containing 200 nM 11-dehydrocorticosterone. Macrophages were incubated overnight in the presence or absence of ∼30 µg/ml copper-oxidized LDL (#BT-910, Biomedical Technologies, Stoughton, MA). Cell media samples were analyzed for cytokine levels via multiplexing immunoassay (#MPXMCYTO-70 K, Millipore, Bedford, MA) using a Luminex 200 System (Austin, TX). Cells were lysed with CelLytic M reagent (#C2978, Sigma, Creamridge, NJ) and total protein content per well was determined by Bradford assay. Cytokine concentrations were normalized to the cellular protein content per well.

### Macrophage RNA Isolation

Peritoneal macrophages were collected by lavage as described in the preceding section. Total RNA was isolated from the resulting cell pellet using 1 ml of Tri-Reagent (#AM9738, Ambion, Inc., Austin, TX) according to the manufacturer's instructions (Manual 9738M RevC). The integrity of RNA samples was assessed under non-denaturing conditions on 2% E-gels (#G5018-02, Invitrogen Inc., Carlsbad, CA).

### RT-PCR Analysis

For whole blood expression analysis, cDNA was synthesized using qScript cDNA SuperMix (#95048, Quanta Biosciences Inc., Gaithersburg, MD). PCR reactions were performed in an ABI7900HT PCR system using Applied Biosystems Taqman Gene Expression Assay Mm00476182_m1 (for 11βHSD1) and Mm01611464_g1 (for ribosomal protein L30, the normalization control) in Taqman Universal PCR Mix (#4304337, Applied Biosystems Inc.). For tissue and peritoneal macrophage expression analyses, cDNA was synthesized with High Capacity cDNA Reverse Transcription Kit (#4368813, Applied Biosystems Inc.) PCR reactions were performed via TaqMan assay using ABI7900HT PCR system. PPIA was used as a control for normalization. For the SYBR® Green assay, total RNA was reverse transcribed according to the manufacturer's instructions (#95048, Quanta Biosciences Inc.). PCR reactions were carried out using Power SYBR® Green PCR Master Mix according to the manufacturer's instructions (#4367659, Applied Biosystems Inc.). Ribosomal protein L30 was used as a control. For both protocols, relative mRNA expression was calculated by fold change using the comparative Ct method (2-ΔΔCt) [22]. Sequences of primer sets used are listed (Table S1).

### Affymetrix Target Labeling, Chip Hybridization and Data Processing

All target labeling reagents and GeneChip® HT One-Cycle Target Labeling kits were purchased from Affymetrix (Santa Clara, CA). Mouse Genome HT_MG-430 arrays were purchased from Affymetrix. Double-stranded complementary DNAs (cDNAs) were synthesized from 1.2 µg total RNA from each tissue sample through reverse transcription with an oligo-dT primer containing the T7 RNA polymerase promoter using the cDNA Synthesis System from Affymetrix. Biotin-labeled cRNAs were generated from the cDNAs and were processed on a Caliper GeneChip Array Station from Affymetrix. Labeled cRNAs were hybridized on Affymetrix Mouse Genome HT_MG-430 arrays. Array hybridization, washing and scanning were performed according to Affymetrix protocol recommendations. Scanned images were subjected to visual inspection and chip quality reports were generated by Expression console (Affymetrix). The image data was processed using the Robust Multichip Average (RMA) method to determine the specific hybridizing signal for each probe set.

### Pathway Analysis

Ingenuity Systems Pathway Analysis (IPA) software version 9.0 (Ingenuity Systems Incorporated; Redwood City, CA; www.ingenuity.com) was used to annotate the Affymetrix probesets for the genes they represent and the functional pathways to which they belong. The canonical pathways and the pathway genes listed in the data analyses were obtained from the Ingenuity IPA systems.

### Data and Statistical Analysis

Data are reported as mean ± standard error of the mean. Statistical analyses were performed using a Student's unpaired t-test, one-way or two-way ANOVA and Tukey's or Dunnett's post-hoc test, as appropriate. Results were considered statistically significant at p≤0.05.

## Results

### 11βHSD1 mRNA Tissue Expression

**Figure 1:**
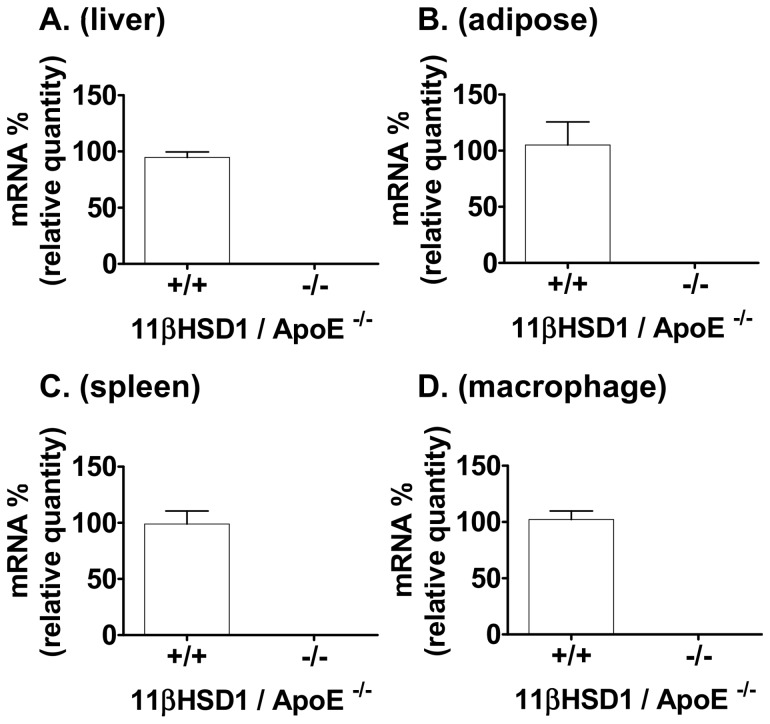
11βHSD1 mRNA expression profiles from 11βHSD1^+/+^/apoE^−/−^ and 11βHSD1^−/−^/apoE^−/−^ mice. Gene expression levels in **A**) liver, **B**) adipose, **C**) spleen and **D**) thioglycollate-elicited peritoneal macrophages. Values normalized to 11βHSD1**^+/+^** levels. Data are from a subset analysis of mixed-sex mice.

Mice deficient in 11βHSD1 were created by targeted disruption of the 11βHSD1 gene locus (Fig. S1). The resulting knockout animals were generated on the apoE^−/−^ background to create the 11β^−/−^/apoE^−/−^ strain. These mice were compared to littermate 11βHSD1 wild-type controls (11β^+/+^/apoE^−/−^). Expression of 11βHSD1 mRNA in selected tissues was determined by quantitative PCR analysis and data are shown in Figure 1A–C. 11βHSD1 gene expression was undetectable in tissues taken from 11β^−/−^/apoE^−/−^ mice vs. littermate wild-type controls. Lack of 11βHSD1 gene expression was also confirmed in thioglycollate-elicited peritoneal macrophages taken from 11β^−/−^/apoE^−/−^ mice vs. control (Fig. 1D). Ablation of the 11βHSD1 gene diminished 11βHSD1 cortisone reductase activity *in vivo* via examination of conversion of an exogenous oral dose of cortisone to cortisol in intact mice (Fig. S2A). In addition, inhibition of reductase activity was also observed in vitro using liver microsomes taken from 11βHSD1 deficient mice (Fig. S2B).

### Atherosclerosis Development

**Figure 2:**
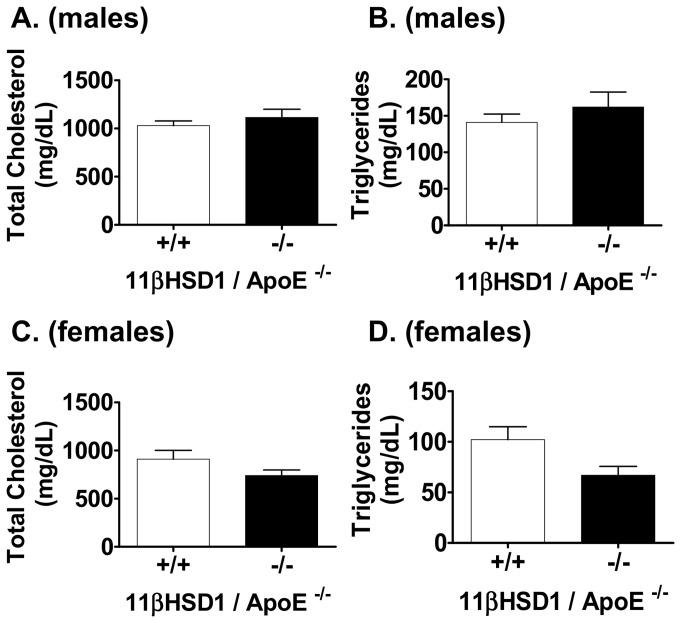
Total plasma cholesterol and triglycerides in fasted 11βHSD1^+/+^/apoE^−/−^ and 11βHSD1^−/−^/apoE^−/−^ mice 14 **weeks post Western diet.** **A**) Plasma total cholesterol and **B**) triglycerides in male mice (n = 23, 11βHSD1^+/+^/apoE^−/−^; n = 13, 11βHSD1^−/−^/apoE^−/−^). **C**) Plasma total cholesterol and **D**) triglycerides in female mice (n = 14, 11βHSD1^+/+^/apoE^−/−^; n = 8, 11βHSD1^−/−^/apoE^−/−^). Data are pooled from three independently run in vivo studies.

To study the effects of 11βHSD1 gene deficiency on atherosclerosis development, mice were fed a Western-type diet starting at eight weeks of age and continued for an additional 14 weeks. All mice gained weight normally (Fig. S3), blood pressures were within a normal range (Fig. S4) and no overt derangements in their basic physiology or behavior were detected. Plasma lipids were measured at the end of the dietary feeding phase and results are shown in Figure 2. All mice displayed elevated plasma total cholesterol and triglycerides as expected for the hyperlipidemic apoE knockout model [23]. Total cholesterol and triglyceride levels were similar between male (Fig. 2A–B) and female (Fig. 2C–D) groups of mice, and between genotypes, indicating no statistically significant effect of 11βHSD1 deficiency on circulating lipid levels. In female mice, however, a non-statistically significant trend towards decreased triglycerides was noted in the 11β^−/−^/apoE^−/−^ group. No significant changes were detected in plasma HDL-cholesterol, non-HDL cholesterol or fasting glucose in any of the groups of mice (Fig. S5).

**Figure 3:**
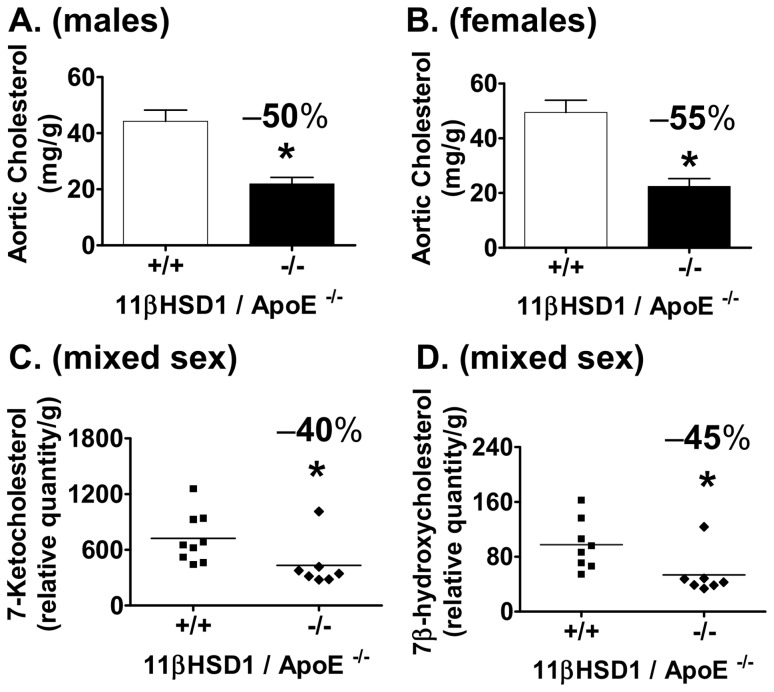
Aortic cholesterol analysis in 11βHSD1^+/+^/apoE^−/−^ and 11βHSD1^−/−^/apoE^−/−^ mice. Total aortic cholesterol mass from each vessel was normalized to delipidated aorta dry weight. **A**) Aortic cholesterol levels in male 11βHSD1^+/+^/apoE^−/−^ (n = 21) and 11βHSD1^−/−^/apoE^−/−^ (n = 15) mice. **B**) Aortic cholesterol levels in female 11βHSD1^+/+^/apoE^−/−^ (n = 15) and 11βHSD1^−/−^/apoE^−/−^ (n = 8) mice. Data are pooled from three independently run in vivo studies. **C**) Aortic 7-ketocholesterol and **D**) 7β-hydroxycholesterol levels in 11βHSD1^+/+^/apoE^−/−^ (n = 9) and 11βHSD1^−/−^/apoE^−/−^ (n = 7) mixed-sex mice. Data are from a single independently run in vivo study. Significance vs. control: *p≤0.05.

**Figure 4:**
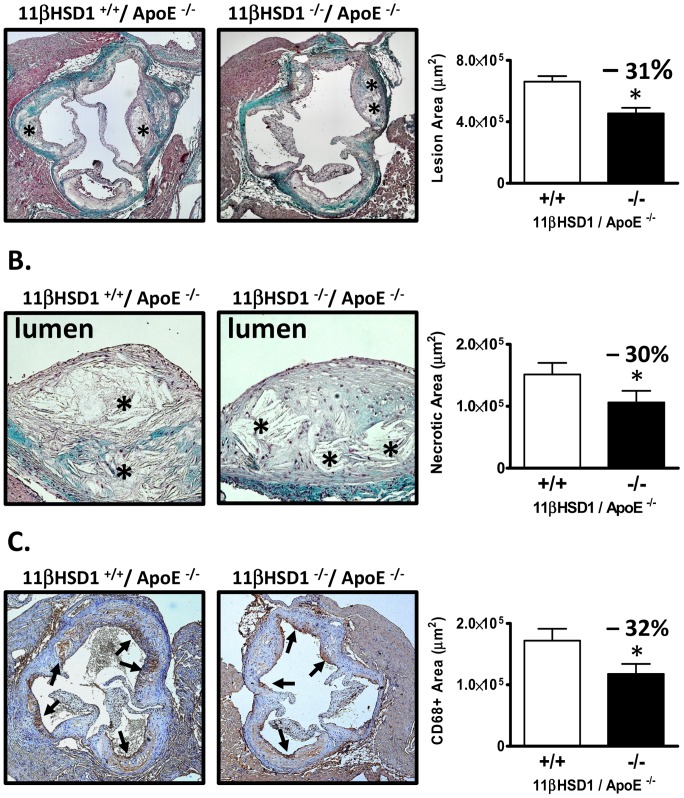
Aortic root atherosclerosis. **A**) Representative trichrome-stained images of lesions in the aortic root and lesion area measurements. Left image: 11βHSD1^+/+^/apoE^−/−^ aortic root. Right image: 11βHSD1^−/−^/apoE^−/−^ aortic root. Asterisks denote regions of acellular necrotic debris. Plot: Lesion area measurements from trichrome-stained images in 11βHSD1^+/+^/apoE^−/−^ (n = 10) and 11βHSD1^−/−^/apoE^−/−^ (n = 9) male mice. **B**) Intra-plaque necrotic areas. Left image: 11βHSD1^+/+^/apoE^−/−^ aortic root plaque. Right image: 11βHSD1^−/−^/apoE^−/−^ aortic root plaque. Asterisks denote necrotic areas within lesions. Acellularity and features of crystalline clefts were used to demarcate necrotic regions. Plot: Necrotic core area measurements from trichrome-stained images in 11βHSD1^+/+^/apoE^−/−^ (n = 10) and 11βHSD1^−/−^/apoE^−/−^ (n = 9) male mice. **C**) Macrophage staining via CD68 immunohistochemistry. Left image: 11βHSD1^+/+^/apoE^−/−^ aortic root. Right image: 11βHSD1^−/−^/apoE^−/−^ aortic root. Arrows denote CD68 positive staining. Plot: CD68 positive stained area measurements in 11βHSD1^+/+^/apoE^−/−^ (n = 10) and 11βHSD1^−/−^/apoE^−/−^ (n = 9) male mice. Significance vs. control: *p≤0.05. Data are pooled from two independently run in vivo studies.

Cholesterol accumulation in the thoracic aortae of mice was evaluated as a measure of atherosclerosis development. In males, aortic total cholesterol levels were reduced by 50% (p<0.01) in 11β^−/−^/apoE^−/−^ mice vs. control (Fig. 3A). Similar effects were observed in female mice (Fig. 3B): 55% (p<0.01) reduction in 11β^−/−^/apoE^−/−^ vs. control, respectively. In an analysis of mice of both genders, aortic 7-ketocholesterol levels were measured and revealed a decrease of 40% in 11β^−/−^/apoE^−/−^ mice vs. control (Fig. 3C). A similar reduction was observed in aortic 7β-hydroxycholesterol (Fig. 3D). Atherosclerosis was also evaluated in the aortic sinus of male mice using the method described by Paigen [19]. Cross-sectional lesion areas were examined following Masson's trichrome staining of serial sections. Representative images and quantitative data (Fig. 4A) show a reduction in aortic root lesion area of 31% in 11β^−/−^/apoE^−/−^ mice vs. control (11β^−/−^/apoE^−/−^: ∼454,000 µm^2^; 11β^+/+^/apoE^−/−^: ∼661,000 µm^2^; p<0.05). Figures 4A and 4B show necrotic core sizes within the intima of aortic root lesions (denoted by asterisks). As shown in Figure 4B, necrotic core area revealed a 30% decrease in 11β^−/−^/apoE^−/−^ mice vs. control (11β^−/−^/apoE^−/−^: ∼106,000 µm^2^; 11β^+/+^/apoE^−/−^: 151,000 µm^2^; p<0.05). Lesional macrophage content measured in the aortic root via CD68 immunohistochemistry is shown in Figure 4C. Analysis of CD68 positive stain area revealed a 32% reduction in 11β^−/−^/apoE^−/−^ mice vs. control (Fig. 4C; p<0.05).

**Figure 5:**
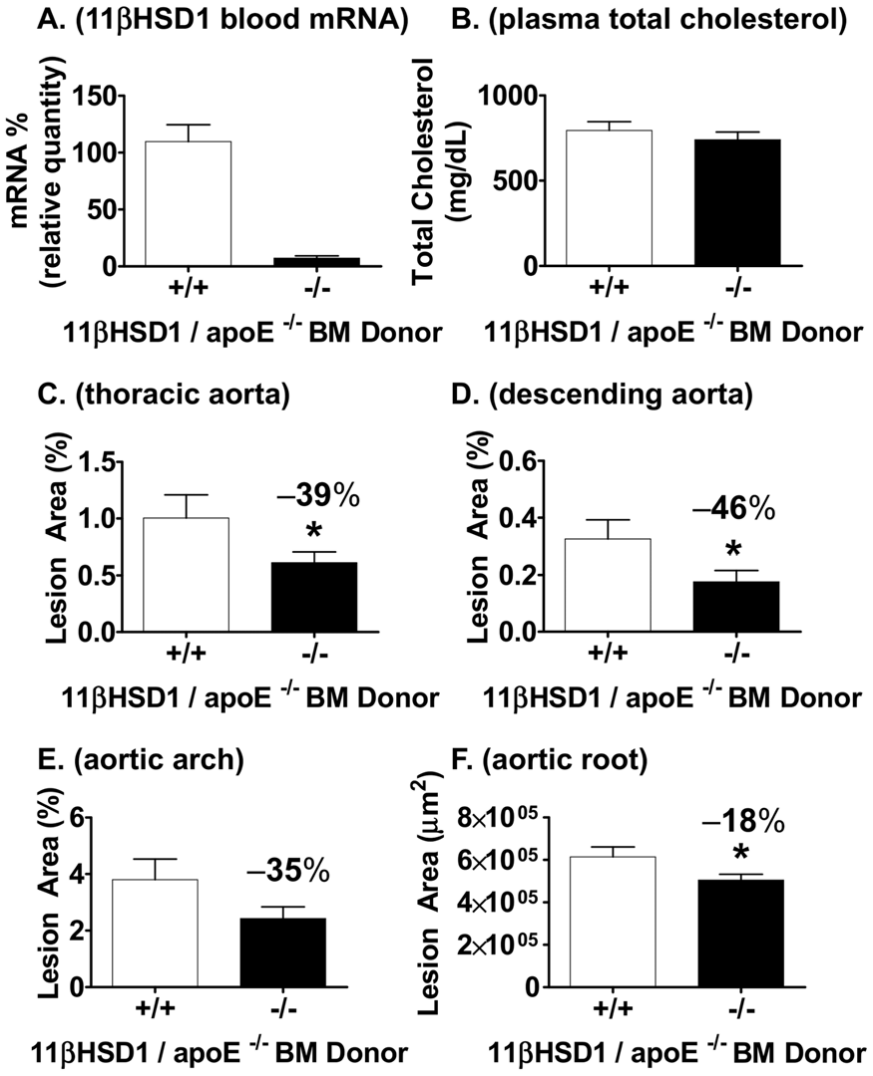
Atherosclerosis development with 11βHSD1-deficient bone marrow reconstitution. **A**) 11βHSD1 gene expression levels in whole blood taken at study termination from apoE knockout mice reconstituted with 11βHSD1^−/−^/apoE^−/−^ (n = 12) or 11βHSD1^+/+^/apoE^−/−^ (n = 13) bone marrow cells. **B**) Plasma total cholesterol levels 12 weeks post Western diet feeding. **C**) Quantification of en face lesion area with oil red O staining in the whole thoracic aorta, **D**) descending aorta and **E**) aortic arch. **F**) Aortic root lesion area. Data are from a single independently run in vivo study. Significance vs. control: *p≤0.05.

The role of leukocyte-derived 11βHSD1 on plaque development was evaluated using the bone marrow transplantation model. Bone marrow-derived stem cells from 11β^−/−^/apoE^−/−^ and 11β^+/+^/apoE^−/−^ littermate control mice were harvested and injected into irradiated apoE-deficient recipient male mice. Following a 4 week engraftment phase, mice were placed onto Western diet for 12 weeks. Confirmation of efficient transplantation was carried out by quantitative PCR analysis of 11βHSD1 mRNA in whole blood from recipient mice. 11βHSD1 mRNA was essentially undetectable in recipients transplanted with 11β^−/−^/apoE^−/−^-derived stem cells vs. control (Fig. 5A). Similar to findings in the Western diet-fed atherosclerosis model (Fig. 2), plasma total cholesterol was similar between both groups of mice (Fig. 5B). En face analysis of the whole thoracic aorta revealed that mice transplanted with 11β^−/−^/apoE^−/−^-derived stem cells developed 39% less atherosclerosis than control mice (Fig. 5C). Plaque area in the descending aorta was reduced by 46% in 11β^−/−^/apoE^−/−^-transplanted mice vs. control (Fig. 5D). Although not statistically significant, a strong trend towards reduced plaque area in the aortic arch of 11β^−/−^/apoE^−/−^-transplanted mice was noted (35% reduction vs. control, p = 0.06; Fig. 5E). In addition, histological analysis of atherosclerosis in the aortic root revealed a reduction in plaque area of 18% vs. control (Fig. 5F).

### In Vivo Inflammatory Cell Migration

**Figure 6:**
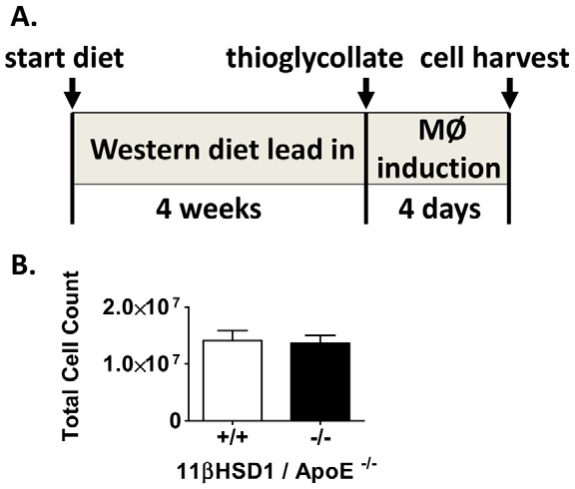
Hyperlipidemic peritonitis model and in vivo cell migration. **A**) Schematic depicting duration of dietary feeding, peritonitis and timing of cell harvest. **B**) Cell counts in lavage fluid from 11βHSD1^+/+^/apoE^−/−^ (n = 13) and 11βHSD1^−/−^/apoE^−/−^ (n = 16) mixed-sex mice.

Given the reduction in macrophage content in aortic root lesions, the results of the bone marrow transplantation studies and the central role of the macrophage in the pathophysiology of atherosclerosis, two important aspects of macrophage function that are known to contribute to plaque formation were further evaluated in vivo: inflammatory cell migration and cholesterol loading capacity. Inflammatory cell migration was evaluated in vivo using Western diet-fed 11β^+/+^/apoE^−/−^ and 11β^−/−^/apoE^−/−^ mice subjected to intraperitoneal thioglycollate challenge. The ∼5 week protocol shown in Figure 6A was followed. Total peritoneal cell counts were measured and no statistically significant differences were detected between groups (Fig. 6B). Phenotyping of the peritoneal lavage showed that greater than 95% of the cells were macrophages (Fig. S6). No significant differences in cell counts for either macrophages or neutrophils were detected between groups. These data suggest that under these experimental conditions, 11βHSD1 does not modulate inflammatory cell migration.

### In Vivo Foam Cell Formation

**Figure 7:**
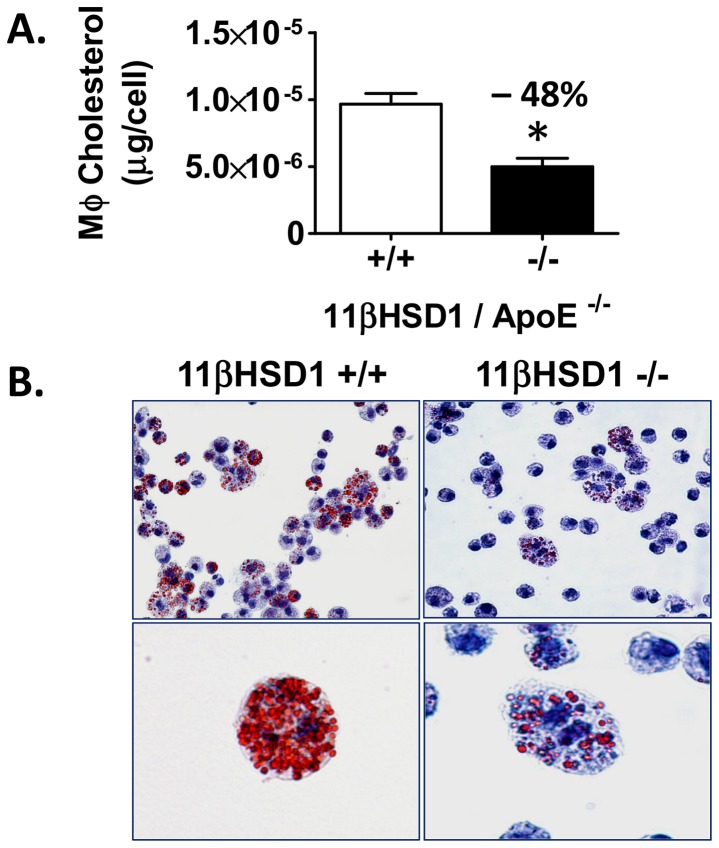
In vivo foam cell analysis. **A**) Peritoneal macrophage total cholesterol mass normalized to cell count from hyperlipidemic 11βHSD1^+/+^/apoE^−/−^ (n = 13) and 11βHSD1^−/−^/apoE^−/−^ (n = 16) mixed-sex mice. **B**) Peritoneal macrophages (MΦ) harvested by lavage from 11βHSD1^+/+^/apoE^−/−^ (left upper and lower images) and 11βHSD1^−/−^/apoE^−/−^ (right upper and lower images) mice stained with oil red O and hematoxylin. Magnification of top images 40X and lower images 100X. Data are pooled from two independently run studies. Significance vs. control: *p≤0.05.

Thioglycollate-elicited peritoneal macrophages were also evaluated for the extent of cholesterol loading following thioglycollate challenge. Prior studies using pro-atherogenic hyperlipidemic mice have shown that thioglycollate-induced peritoneal macrophages can accumulate lipid sterols in vivo and possess a phenotype suggestive of foam cells [24]–[25]. Following the protocol shown in Figure 6A, peritoneal macrophages harvested from 11β^+/+^/apoE^−/−^ and 11β^−/−^/apoE^−/−^ mice were subjected to organic extraction of cellular lipids followed by quantification of total cholesterol. As shown in Figure 7A, macrophage cholesterol content was reduced by 48% in 11β^−/−^/apoE^−/−^ mice vs. control. Consistent with these findings, microscopic evaluation of peritoneal macrophages stained with oil red O revealed higher levels of lipid droplets in macrophages from 11β^+/+^/apoE^−/−^ than 11β^−/−^/apoE^−/−^ (Fig. 7B).

### Gene Expression Profiling Analysis

Affymetrix gene expression profiling was carried out with thioglycollate-elicited peritoneal macrophages from 11β^+/+^/apoE^−/−^ and 11β^−/−^/apoE^−/−^ mice. Gene expression profiles were compared by Two-Way ANOVA using Partek® Discovery Suite software. Only the Affymetrix probe sets that displayed a maximum anti-log RMA signal intensity of greater than 16 were considered for post hoc analyses. Differential changes in gene expression levels ranged from ∼+2.3 fold for up-regulated genes (428 genes) and ∼−3.8 fold for down-regulated genes (434 genes). Interestingly, given the observation of decreased in vivo cholesterol loading with 11βHSD1 deficiency, there were no significant changes in transcript levels of genes associated with oxidized LDL scavenging/uptake in macrophages, for example, scavenger receptor A1(SR-A1), A2(SR-A2), B1(SR-B1) and thrombospondin receptor CD36. Similarly, there were no transcript level changes in genes classically associated with macrophage cholesterol homeostasis: acetyl-CoA acetyltransferase 1 (ACAT1), cholesteryl ester hydrolase (CEH), nuclear hormone receptors LXRα and LXRβ and ABC transporter proteins ABCA1 and ABCG1. However, a significant 1.8 fold decrease (p = 0.001) in toll-like receptor 4 (TLR 4) gene expression was detected in 11β^−/−^/apoE^−/−^ mice vs. control. Further inspection of the TLR gene family revealed significant decreases in TLR 1, 3, 4, 8 and 13 (Table 1) in 11β^−/−^/apoE^−/−^ mice. These data indicate that 11βHSD1 gene knockout leads to significant down regulation of selected TLRs in peritoneal macrophages.

**Table 1:** Differentially expressed genes related to toll-like receptor signaling in peritoneal macrophages from Western-diet fed 11β^+/+^/apoE^−/−^ and 11β^−/−^/apoE^−/−^ mice.

Gene Symbol	Gene Name	11β^−/−^/vs. control	
		fold change	p value
TLR1	Toll-like receptor 1	−1.2	1E-02
TLR3	Toll-like receptor 3	−1.3	1E-02
TLR4	Toll-like receptor 4	−1.8	2E-03
TLR8	Toll-like receptor 8	−1.2	1E-03
TLR13	Toll-like receptor 13	−1.2	3E-03
Edem3	ER degradation enhancer, mannosidase alpha-like 3	−1.6	1E-03
Jak2	Janus kinase 2	−1.3	3E-02
Map3k2	Mitogen-activated protein kinase kinase kinase 2	−1.5	3E-03
Traf3ip3	TRAF3 interacting protein 3	−1.3	1E-03
Traf5	TNF receptor-associated factor 5	−1.3	7E-03

Toll-like receptors and associated signal transduction pathway genes.

**Figure 8:**
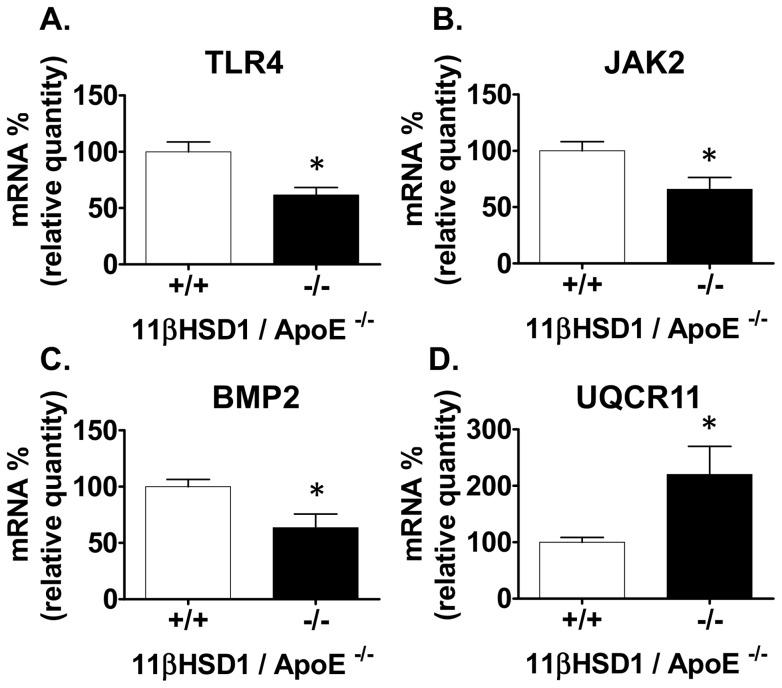
Gene expression levels by RT-PCR from peritoneal macrophages. **A**) TLR4, **B**) JAK2, **C**) BMP2 and **D**) UQCR11 mRNA profiling. Expression data from 11βHSD1^+/+^/apoE^−/−^ (n = 6) and 11βHSD1^−/−^/apoE^−/−^(n = 5) mice are shown in each plot. Symbols indicate significance vs. control: *p≤0.05.

Further analysis of peritoneal macrophage microarray data revealed down regulation of genes associated with TLR signal transduction. As shown in Table 1, TNF receptor-associated factor 5 (TRAF5) and TRAF3 interacting protein 3 (TRAF3IP3) were decreased in 11β^−/−^/apoE^−/−^ mice vs. control. Other downstream TLR signal transduction pathway genes including Map3k2 and JAK2 were also down regulated relative to control (Table 1). In addition, endoplasmic reticulum degradation enhancer, mannosidase alpha-like 3 (Edem3) mRNA levels were also down-regulated 1.6 fold in 11β^−/−^/apoE^−/−^ vs. control. Real-time quantitative PCR of several key genes from the microarray studies confirmed the magnitude and direction of changes observed by microarray analysis (Fig. 8).

An evaluation of significantly affected biological pathways with 11βHSD1 gene deficiency was carried by Ingenuity Pathway Analysis (IPA). IPA revealed reductions in genes associated with inflammatory/immune function, suggesting that the overall inflammatory state of 11βHSD1-deficient macrophages was attenuated (Table 2). Inflammatory pathways that were attenuated included; chemokine signaling, JAK/Stat signaling, pattern recognition receptor signaling (Table S2A–C), NF-κB signaling (Table S2D) and glucocorticoid receptor signaling (Table S2E). In addition, IPA revealed significant differential expression of genes associated with mitochondrial function/energy generation: oxidative phosphorylation was the most impacted pathway (Table 2 and Table S3). Mitochondrial genes involved in the electron transport chain (i.e., ATP production), NADH dehydrogenase, ubiquinol-cytochrome C reductase and ATP synthase were significantly up-regulated in 11β^−/−^/apoE^−/−^ mice.

**Table 2:** Ingenuity canonical pathways significantly impacted in peritoneal macrophages from Western-diet fed 11β^+/+^/apoE^−/−^ and 11β^−/−^/apoE^−/−^ mice.

Ingenuity Canonical Pathway	11β^−/−^/vs. control		
	p value	ratio	
Oxidative Phosphorylation	5.01E-14	30%	
Ubiquinone Biosynthesis	1.00E-11	26%	
Mitochondrial Dysfunction	1.00E-10	23%	
EIF2 Signaling	2.45E-09	23%	
Protein Ubiquitination Pathway	2.88E-06	19%	
Estrogen Receptor Signaling	4.37E-06	23%	
Purine Metabolism	1.05E-04	12%	
NRF2-mediated Oxidative Stress Response	1.91E-04	18%	
Pyrimidine Metabolism	4.47E-04	13%	
Chemokine Signaling	1.58E-03	22%	
Glucocorticoid Receptor Signaling	2.88E-03	14%	
JAK/Stat Signaling	3.31E-03	22%	
NF-κB Signaling	4.27E-03	17%	
Apoptosis Signaling	9.55E-03	18%	
PI3K/AKT Signaling	1.07E-02	15%	
Activation of IRF by Cytosolic Pattern	1.15E-02	18%	
Recognition Receptors: Aldosterone Signaling in Epithelial Cells	7.24E-02	13%	

### Cytokine Secretion with Pro-Atherogenic TLR Ligand Challenge

**Figure 9:**
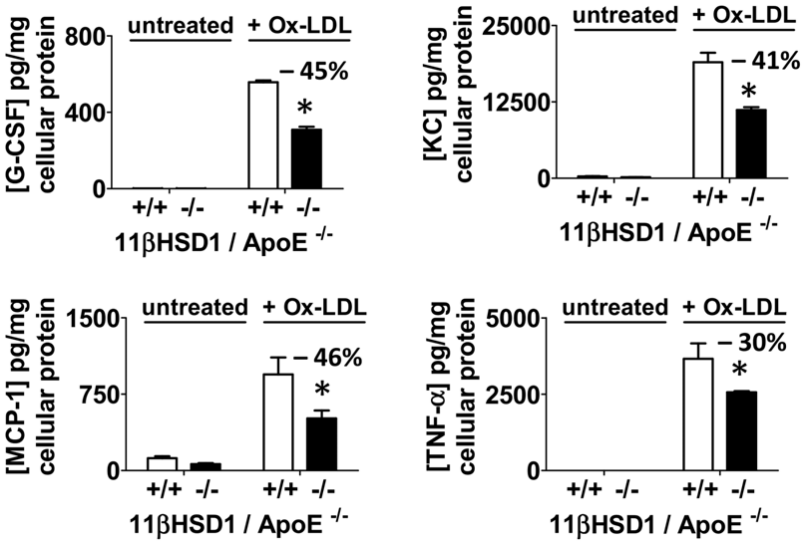
Cytokine secretion following in vitro stimulation with oxidized LDL. Cytokine protein levels detected in cell culture media by protein multiplex analysis following overnight exposure of peritoneal foam cells to ∼30 µg/ml of oxidized LDL (Ox-LDL) vs. untreated controls. Peritoneal foam cells were harvested from Western diet-fed 11βHSD1^+/+^/apoE^−/−^ (+/+) and 11βHSD1^−/−^/apoE^−/−^ (−/−) mice. G-CSF, KC, MCP1 and TNF-α levels were normalized to total cellular protein content. Cell culture media contained 200 nM 11-dehydrocorticosterone throughout the course of the experiment. Significance vs. control: *p≤0.05.

Cytokine release from cultured thioglycollate-elicited peritoneal macrophages from Western diet-fed 11β^+/+^/apoE^−/−^ and 11β^−/−^/apoE^−/−^ mice was examined by antibody array analysis following stimulation with oxidized LDL. Incubation conditions and challenge with oxidized LDL occurred in the presence of the 11βHSD1 substrate 11-dehydrocorticosterone. As shown in Figure 9, treatment with oxidized LDL stimulated the release of several pro-inflammatory cytokines from cultured cells of both strains of mice vs. untreated control, including granulocyte colony stimulating factor (G-CSF), keratinocyte-derived chemokine (KC; murine homologue of IL-8), monocyte chemotactic protein-1 (MCP-1) and tumor necrosis factor-alpha (TNF-α). Release of these cytokines into cell culture media was reduced with 11βHSD1 deficiency (Fig. 9). Significant reductions in GCSF, KC and MCP-1 ranged from 41-46% vs. control. Release of TNF-α by 11βHSD1-deficient peritoneal macrophages was reduced by 30% vs. control. Seven additional cytokines that were measured yielded variable responses in replicate studies: IL-1β, IL-6, IP-10, MIP-1A, MIP-1B, MIP-2, RANTES (data not shown). In addition, cytokine levels for peritoneal macrophages not challenged with oxidized LDL were at or below the detection limit of the assay.

## Discussion

Treatment of hyperlipidemic mice with pharmacological inhibitors of 11βHSD1 has been shown to reduce plaque in the presence of reduced plasma lipids. For example, a study in the Western diet-fed apoE knockout mouse showed that eight weeks of treatment with a selective 11βHSD1 inhibitor decreased aortic cholesterol burden by 84% vs. vehicle control [17]. Plaque lowering was observed along with reductions in circulating total cholesterol (∼30%), triglycerides (∼60%) and glucose (44%). Similarly, a study performed in a variant of the LDLr knockout mouse, that exhibits characteristics of the metabolic syndrome, showed that four weeks of treatment with carbenoxolone, a pan-11βHSD inhibitor, decreased aortic root plaque size by ∼30% vs. vehicle control [18]. In addition, carbenoxolone treatment significantly improved plasma lipid profiles, enhanced VLDL clearance rates and lowered fasting plasma insulin levels. Full interpretation of the latter study, however, is limited by the non-selectivity of carbenoxolone, which inhibits both 11βHSD1 and 11βHSD2 with similar affinity. While these reports were important in first demonstrating the intriguing potential of 11βHSD1 inhibitors to ameliorate atherosclerosis, the results could not distinguish whether the reduction in plaque burden was due to a direct effect on the vessel wall, an indirect effect via improvement in the lipid profile and/or glucose metabolism, changes in blood pressure, or a combination of the above.

In order to elucidate the physiological and molecular mechanisms underlying the anti-atherosclerotic effect of 11βHSD1 inhibition, the studies described herein were performed. The results indicate that inactivation of 11βHSD1 can positively impact aortic plaque by decreasing i) aortic cholesterol and 7-oxysterol accumulation, ii) aortic root plaque area iii) necrotic core intimal area and iv) CD68-positive macrophage inflammation. These data support a rapidly evolving concept that inhibition of 11βHSD1 can ameliorate atherosclerotic disease via direct effects at the arterial wall, without impacting plasma lipids or other metabolic parameters [3]. Furthermore, bone marrow transplantation experiments revealed that the absence of leukocyte-specific 11βHSD1 reduces plaque burden, suggesting direct involvement of monocyte/macrophage 11βHSD1 in atherosclerosis development. Upon closer inspection of macrophage phenotype, the observed downregulation of several TLRs, including TLR4 and the attenuated cytokine response to oxidized LDL challenge suggests a key role of 11βHSD1 in modulating TLR interactions with pro-atherogenic ligands.

Analyses of cholesterol levels in the thoracic aortae of mice revealed significant decreases in cholesterol (Fig. 3A–B) and 7-oxysterols (Fig. 3C–D) with 11βHSD1 deficiency. Oxysterols such as 7-ketocholesterol and its metabolite 7β-hydroxycholesterol are among the most abundant oxysterols in human foam cell macrophages and advanced human lesions [10]–[11]. Aortic oxysterols of mice of both genotypes revealed detectable amounts of 7-ketocholesterol and 7β-hydroxycholesterol. 11β^−/−^/apoE^−/−^ mice had significantly lower absolute levels of 7-ketocholesterol and 7β-hydroxycholesterol than 11β^+/+^/apoE^−/−^ control mice (−40% and −45%, respectively, Fig. 3C–D). Studies in culture have shown that mouse peritoneal macrophages loaded with 7-ketocholesterol-enriched LDL particles are impaired in their ability to efflux cholesterol to apoA-I acceptor particles [26]. Thus, the reduction in aortic 7-ketocholesterol levels observed with decreasing 11βHSD1 gene levels may promote an improvement in the ability of macrophages to unload cholesterol and thereby attenuate foam cell formation in the vessel wall.

Histological analyses of structure and composition of the atherosclerotic plaques present in the aortic root revealed a 30% reduction in necrotic core area with 11βHSD1 gene deficiency (Fig. 4B). We performed CD68 immunohistochemistry to determine whether changes in macrophage activation state and/or macrophage numbers could account for the reductions in necrotic core area. CD68 is a useful marker of both macrophage number and also the state of macrophage activation in the context of the atherosclerotic plaque. CD68 is highly upregulated as macrophages take up oxidized lipids in the process of becoming foam cells [27]. Aortic root samples from homozygous 11βHSD1 knockout mice had 32% less staining for CD68 than samples taken from control mice. We asked whether this reduction was due to i) differences in circulating monocyte levels between groups, ii) impairment in monocyte infiltration or iii) a potential change in cell surface CD68 expression due to a reduction in foam cell formation.

The first possibility was addressed by analyzing blood samples from 11β^+/+^/apoE^−/−^ and 11β^−/−^/apoE^−/−^ mice (naïve and thioglycollate injected): no differences in monocyte or total leukocyte counts were detected between groups (Fig. S7). To address the second possibility, macrophage infiltration capacity was tested via the ability of macrophages to migrate into the peritoneum in response to thioglycollate challenge. The data presented in Figure 6B indicate that 11βHSD1 gene deficiency does not modulate inflammatory cell migration into the peritoneum within the context of the experimental design. Cell counts are similar regardless of the genotype. Similar results have been reported in the literature for independently derived 11βHSD1 knockout mice [28]. While migration into the peritoneum is not precisely the same as migration into the vessel wall, it does indicate that there is no major change in macrophage migration capacity in response to an inflammatory stimulus. It therefore suggests that 11βHSD1-mediated regulation of inflammatory cell migration/infiltration is not likely to have a substantial effect on CD68 staining and atherosclerosis in this model.

Given this conclusion, the third possibility was tested. Mice of both genotypes were placed on a Western diet for 4 weeks. At the end of this period, they were subjected to a peritoneal thioglycollate challenge to elicit recruitment of macrophages into the peritoneum and stimulate foam cell formation with hyperlipidemia. Staining and microscopic evaluation of macrophages showed a distinct foamy appearance and the presence of intracellular lipid droplets due to the accumulation of cholesteryl ester (Fig. 7B). Quantification of cellular lipid content showed that the macrophages isolated from 11β^−/−^/apoE^−/−^ mice had 48% less cholesterol than macrophages isolated from control mice (Fig. 7A). It therefore is likely that reduced CD68 levels in aortic root plaque of 11βHSD1 knockout mice is at least partially related to the reduced foam cell-like character of macrophages isolated from these animals leading to either downregulation of CD68 expression or a decrease in total foam cell area. Regardless, the reduction in aortic CD68 staining is indicative of a decrease in the foam cell state generated by macrophages in the plaque.

Mouse bone marrow transplantation studies have been used to study the role of leukocyte-associated genes on the pathophysiology of atherosclerosis. The results of the analysis described herein clearly indicate the involvement of leukocyte associated-11βHSD1 in atherosclerosis development. In these studies, plaque burden in the thoracic aorta was reduced 39% with 11βHSD1 deficiency vs. control (Fig. 5C). These changes are consistent with findings obtained in the diet-induced atherosclerosis experiments described in this report (50% reduction in aortic cholesterol content of male 11β^−/−^/apoE^−/−^ mice; Fig. 3A). In both cases, total circulating cholesterol levels were similar between groups despite differences in 11βHSD1 expression levels (compare Fig. 2A and Fig. 5B). Thus, both atherosclerosis studies provide direct evidence that 11βHSD1 deficiency can attenuate vessel wall atherosclerosis independent of changes in plasma cholesterol. The observation that monocyte/macrophage-specific 11βHSD1 deficiency is associated with decreased plaque burden may relate, in part, to macrophage-associated 11βHSD1 function. Foam cells are largely derived from circulating monocytes that differentiate into pro-inflammatory macrophages upon infiltration into the vascular subendothelium. Studies in culture with human monocytes have shown that monocyte to macrophage differentiation is a strong stimulus for 11βHSD1 expression [29]. Moreover, 11βHSD1 expression is completely absent in pre-differentiated human monocytes, whereas macrophages differentiated with pro-inflammatory agents (i.e., LPS, cytokines) express high levels of 11βHSD1 [29]. Although we cannot rule out the impact of other immune cells lacking 11βHSD1 in the bone marrow transplantation studies (i.e., lymphoid and other myeloid types), the association between 11βHSD1 and macrophage function is likely.

The critical observations that leukocyte-specific 11βHSD1 deficiency reduces plaque burden and in vivo cholesterol accumulation in peritoneal macrophages is reduced in 11β^−/−^/apoE^−/−^ mice suggest that inhibition of 11βHSD1 can modulate an important function of the macrophage central to atherosclerosis pathophysiology. Prior studies by other investigators have shown that hyperlipidemic peritoneal macrophages possess phenotypic and functional attributes of lesional macrophages and appear to be relevant surrogates of macrophages present in atheroma [25] [24]. Based on those previous findings, microarray studies of in vivo peritoneal foam cells were performed to elucidate the potential pathways that drive reduced foam cell formation and atherosclerosis in 11βHSD1 deficient mice. While many differences in the gene expression profile were noted, of particular interest to this discussion is the observation that ablation of the 11βHSD1 gene resulted in downregulation of several toll-like receptors (TLRs) including TLR 1, 3, 4, 8 and 13. In particular, the mRNA for TLR4 was downregulated approximately two fold as confirmed by quantitative PCR. Moreover, the impact of decreased TLR transcript levels translated to a diminished functional cytokine response following challenge with oxidized LDL, a known pro-inflammatory and pro-atherogenic ligand that binds TLR4.

TLR4 is expressed by foam cells, is present in human coronary artery plaque, is up regulated by oxidized LDL and, most relevant to the results presented herein, is involved in macrophage-mediated lipid accumulation [30]–[33]. Indeed, Higashimori et al. [34] showed that TLR4/apoE double knockout mice accumulated ∼75% less cholesterol in the aortic arch vs. control without changes in circulating lipids. Down regulation of the TLR family of genes, including TLR4, in 11βHSD1-deficient macrophages therefore represents one likely mechanistic explanation for the reduced cholesterol accumulation observed in our *in vivo* foam cell experiments and the reduction in atherosclerosis without a concomitant change in the plasma lipid profiles. The attenuated cytokine response from 11βHSD1-deficient macrophages following TLR4 agonist stimulation with oxidized LDL suggests that 11βHSD1 is important for potentiating TLR4-dependent activation and signaling in the setting of atherosclerosis. This observation is without precedent and suggests a significant interaction between foam-cell associated TLRs and 11βHSD1 function. Interestingly, studies in culture using rat peritoneal macrophages have shown that exposure to corticosterone can modulate TLR2 mRNA levels without directly impacting TLR4 expression [35]. These results suggest a more complex interaction with TLR4 beyond regulation of cortisol/corticosterone levels by 11βHSD1. Additional studies are needed to fully understand this relationship.

To discern additional mechanistic hypotheses for the anti-atherosclerotic effect of the 11βHSD1 gene knockout, the microarray data were further analyzed via an Ingenuity Pathway Analysis (IPA). The results of this analysis (Table S2) revealed significant modulation of genes related to inflammatory function (chemokine signaling, JAK/STAT signaling, pattern recognition, NF-κB signaling, and glucocorticoid receptor signaling) in 11β^−/−^/apoE^−/−^ peritoneal macrophages vs. control.

The pattern for chemokine, NF-κB and JAK/STAT signaling, combined with the downregulation of the pattern recognition receptors is consistent with an anti-inflammatory response to 11βHSD1 gene ablation and the relative anti-atherosclerotic phenotype observed in 11β^−/−^/apoE^−/−^ mice. On the other hand, the broad downregulation in the glucocorticoid signaling pathway is not, on the surface, consistent with an anti-atherosclerotic effect given the known anti-inflammatory effects of pharmacological glucocorticoid therapy. One possibility to explain this discrepancy is that the decrease in signaling via the glucocorticoid receptor (GR) is balanced or dominated by a similar decrease in signaling via the aldosterone-mineralocorticoid receptor pathway. It is well known that aldosterone is a pro-inflammatory agent in macrophages, [36] an effect that can be blocked with mineralocorticoid receptor (MR) antagonists such as spironolactone or eplerenone. In the vasculature, aldosterone infusion has been shown to stimulate neo-intimal hyperplasia and vascular remodeling following endothelial injury [37]. Also, it is well known that cortisol has a higher affinity for the MR than it does for the GR. Therefore, it is possible that reducing intracellular glucocorticoid tone via the 11βHSD1 knockout reduces pro-inflammatory signaling via the MR to a greater extent than it reduces the anti-inflammatory signaling through the GR. Support for this concept comes from studies in 11βHSD2/apoE double knockout mice, where loss of inactivation of corticosterone to 11-dehydrocorticosterone accelerates plaque development and intimal inflammation [38]. In the same study, MR antagonism with eplerenone reduced plaque development despite an increase in tissue glucocorticoids. Importantly, treatment with eplerenone did not lower blood pressure, indicating that aldosterone function was not significantly impacted. Similar to these findings, our studies did not reveal an impact on blood pressure with 11βHSD1 deficiency (Fig. S4). Thus the anti-atherosclerotic activity was likely mediated by inhibition of corticosterone-MR interactions, further supporting the concept that reduction in glucocorticoid tone at the MR is anti-atherosclerotic.

The IPA analysis also revealed significant differential changes in expression of genes related to mitochondrial oxidative phosphorylation (Table 2 and Table S3). Most of these genes were up regulated. These findings are intriguing and suggest that ATP production is enhanced in thioglycollate-elicited peritoneal macrophages deficient in 11βHSD1. The increase in oxidative phosphorylation genes in 11βHSD1-deficient macrophages may relate to decreases in intracellular glucocorticoid tone that promote a less inflammatory macrophage phenotype, as suggested by our inflammation-related gene profiling data. Interestingly, published studies by Vats et al. showed that polarization of macrophages to the less inflammatory M2 type using IL4 stimulation induced robust increases in genes associated with mitochondrial energy metabolism, including those involved in uptake and oxidation of fatty acids [39]. These changes in oxidative metabolism genes translated to increases in fatty acid uptake and β-oxidation activity by M2 macrophages [39]. By contrast, in the same report, pro-inflammatory M1 macrophages were shown to preferentially utilize glucose and suppress β-oxidation. The preference for glucose and glycolytic ATP and concomitant arrest of oxidative phosphorylation by pro-inflammatory M1 macrophages has also been demonstrated in murine J774 macrophages [40]–[41]. Taken together, these studies indicate that oxidative phosphorylation is inherently linked to the less inflammatory phenotype associated with M2 macrophages. While there were no significant differences in other well-recognized M2 marker genes (e.g., arginase I, mannose receptor C-type I) relative to 11βHSD1 gene expression levels in our microarray studies (data not shown), the increase in oxidative phosphorylation genes may regulate overall macrophage activities that favor attenuated plaque development.

Besides reducing the cholesterol content within the vessel wall, reduction in macrophage foam cell formation can also reduce the progression from simple foam-cell laden fatty streaks to lesions with necrotic cores. It is generally accepted that accumulation of cholesterol and oxidized lipids drives foam cell formation, the subsequent sequelae of pro-inflammatory changes and eventually, apoptotic and/or necrotic cell death [42]–[44]. Applying these observations to the current data, evaluation of plaque phenotype in the current study revealed a significant reduction in average necrotic area in the aortic root of 11β^−/−^/apoE^−/−^ mice (−30% vs. control; Fig. 4A–B).

We therefore propose a model whereby the absence of 11βHSD1 attenuates macrophage foam cell formation via downregulation of the toll-like receptors. This in turn reduces uptake of cytotoxic levels of sterols and oxidized lipids, lessening the insult to the cell and diminishing the propensity of macrophages to proceed to necrosis. As fewer macrophages enter necrosis, there will be reduced substrate for necrotic core formation. Besides directly reducing necrotic core formation, reducing macrophage necrosis will also reduce the pro-inflammatory signal that stimulates further macrophage infiltration. This would result in prolongation of the rate at which the plaque matures to the point of being pathological.

In addition, given recent advances in the understanding of the natural history of macrophages in atherogenesis, [45] it is also possible that reducing macrophage foam cell formation may not just reduce necrosis, but also enhance clearance of necrotic cells (efferocytosis) as part of the process of resolution of inflammation [46]. It has been reported that impaired clearance of dying cells in atherosclerotic plaque may contribute to necrotic core formation [47] [46]. It is possible that the 11βHSD1 knockout, by reducing the extent of macrophage foam cell formation, allows macrophages to proceed down a pathway leading to enhanced efferocytosis. Our data indicate i) a switch to oxidative phosphorylation for increased ATP production and ii) overall attenuation of pro-inflammatory pathways by 11βHSD1-deficient macrophages, suggesting the development of a macrophage phenotype that is more resolution-competent and less inflammatory [39]. The resulting removal of necrotic material from the plaque would provide a protective function to guard the surrounding intimal environment from harmful exposure to pro-atherogenic reactive oxygen species, pro-inflammatory cytokines, pro-thrombotic proteins, extracellular matrix degrading enzymes and other factors that contribute to the vicious cycle of atherogenesis leading to plaque instability. The end result of this would be a slowing of atherogenesis coupled with enhanced resolution of inflammation, leading to a decrease in overall plaque area with the potential for overall improvement in the structure and function of the vessel wall.

The findings reported herein demonstrate that 11βHSD1 inhibition can attenuate development of atherosclerotic disease directly at the vessel wall without necessarily affecting plasma lipid profiles. Decreasing cholesterol loading in macrophages via downregulation of toll-like receptors offers one plausible mechanistic explanation for atheroprotection observed in this model. It is clear that additional investigation at the molecular level and via pharmacological intervention is required to confirm the hypotheses generated by these *in vivo* studies.

The utility of 11βHSD1 inhibition as a treatment for atherosclerosis is becoming increasingly evident. At present, however, efforts to develop 11βHSD1 inhibitors in the clinic have primarily focused on management of type 2 diabetes [48]. Our results suggest that direct therapeutic targeting of the vessel wall with 11βHSD1 inhibitors may also offer benefit. This raises the intriguing possibility of a single therapeutic agent that can address both the metabolic and macrovascular pathologies associated with type 2 diabetes and the metabolic syndrome. Clinical studies with appropriate pharmacological agents are required to confirm this exciting potential.

## Supporting Information

Figure S1 **Targeted disruption of the 11βHSD1 gene locus.** **A**) Targeting strategy used to disrupt the 11βHSD1 locus. Homologous recombination (represented by X) between the targeting vector and the 11βHSD1 gene results in the replacement of exons 4 and 5 with the selection cassette. The 11βHSD1 targeting vector was derived using the Lambda KOS system. The Lambda KOS phage library, arrayed into 96 superpools, was screened by PCR using exons 4 and 5-specific primers Hsd-1 (5′-AGGTAGTGTCTCGCTGCCTT-3′) and Hsd-3 (5′-CTTCGCACAGAGTGGATGTC-3′). PCR-positive phage superpools were plated and screened by filter hybridization using the 306 bp amplicon derived from primers Hsd-1 and Hsd-3 as a probe. Three pKOS genomic clones, pKOS-63, pKOS-73 and pKOS-82, were isolated from the library screen and confirmed by sequence and restriction analysis. The yeast selection cassette and pKOS-73 were co-transformed into yeast. Clones that had undergone homologous recombination to replace a 408 bp region containing exon 4 and exon 5 with the yeast selection cassette were isolated. The yeast cassette was subsequently replaced with a LacZ/Neo selection cassette to complete the 11βHSD1 targeting vector. The Not I linearized targeting vector was electroporated into 129/SvEv^Brd^ (Lex-1) ES cells. G418/FIAU resistant ES cell clones were isolated, and correctly targeted clones were identified and confirmed by Southern analysis using a 328 bp 5′ external probe (9/10), generated by PCR using primers Hsd-9 (5′-CAATGCATCCATGCGCCTGAA-3′) and Hsd-10 (5′-AGAGACCAGACATTAGGACAC-3′) and a 607 bp 3′ internal probe (Neo5/2), amplified by PCR using primers Neo-5 (5′-GGCAGCGCGGCTATCGTG-3′) and Neo-2 (5′-TCAGAAGAACTCGTCAAG-3′). **B**) Southern hybridization indicating proper gene targeting in the embryonic stem cell clones. Clones 1A10 and 1D2 were selected for blastocyst injections, as denoted by the asterisk symbols. Lex-1 represents untransfected embryonic stem cell DNA.
(TIF)

Figure S2 **Glucocorticoid conversion studies.** **A**) 11βHSD1^+/+^/apoE^−/−^ (n = 4) and 11βHSD1^−/−^/apoE^−/−^ (n = 4) were given a single oral administration of cortisone (10 mg/kg) and plasma cortisol was measured at 0, 30, 60 and 120 minutes post cortisone dosing. Area under the curve was calculated for each profile and results are shown in the bar graph. **B**) Liver microsomes from11βHSD1^−/−^ (n = 7) and 11βHSD1^+/+^ (n = 8) littermate controls were used to evaluate dehydrogenase activity in vitro. For the preparation of microsomes, freshly harvested livers were immediately homogenized and processed by differential centrifugation. The microsomal pellet for each specimen was resuspended and a 100 µg sample incubated with 1 µM 11-dehydrocorticosterone. Corticosterone levels were measured by LC-MS. Values are mean ± SE.
(TIF)

Figure S3 **Animal body weights during Western diet feeding phase of atherosclerosis studies.** **A**) Males. **B**) Females. Circles: 11βHSD1^+/+^/apoE^−/−^; squares: 11βHSD1^−/−^/apoE^−/−^. Values are mean ± SE.
(TIF)

Figure S4 **Blood pressures in hyperlipidemic 11βHSD1^+/+^/apoE^−/−^ and 11βHSD1^−/−^/apoE^−/−^ mice.** **A**) Systolic, **B**) diastolic and **C)** mean arterial pressure (MAP) in mixed sex 11βHSD1^+/+^/apoE^−/−^ (n = 8) and 11βHSD1^−/−^/apoE^−/−^ (n = 7) mice. Blood pressures were measured in conscious mice using a non-invasive computerized tail cuff system (CODA Non-Invasive Blood Pressure Monitor, Kent Scientific Corporation, Torrington, CT). Mice were conditioned to tail cuff instrumentation over several days to control for stress. As a normolipidemic reference, blood pressures were also measured in chow-fed male C57BL/6 mice (n = 7; noted as “C57” in bar graph). Data for individual animals represent the average of at least 5 acquisitions. Values are mean ± SE.
(TIF)

Figure S5 **Fasting plasma HDL-C, non-HDL-C and glucose levels in 11βHSD1^+/+^/apoE^−/−^ and 11βHSD1^−/−^/apoE^−/−^ mice following 14 weeks of Western diet.** Males (n = 23, 11βHSD1^+/+^/apoE^−/−^; n = 13, 11βHSD1^−/−^/apoE^−/−^): **A**) HDL-C, **C**) non-HDL-C and **E**) glucose. Females (n = 14, 11βHSD1^+/+^/apoE^−/−^; n = 8, 11βHSD1^−/−^/apoE^−/−^): **B**) HDL-C, **D**) non-HDL-C and **F**) glucose. Values are mean ± SE.
(TIF)

Figure S6 **Differential phenotyping of post-thioglycollate peritoneal lavage inflammatory cells.** Cells were identified as **macrophage, neutrophil, lymphocyte or mast cell** following chemical staining and microscope evaluation. White bars (n = 10): 11βHSD1^+/+^/apoE^−/−^; black bars (n = 11): 11βHSD1^−/−^/apoE^−/−^. Values are mean ± SE.
(TIF)

Figure S7 **White blood cell and monocyte levels in blood from naïve and thioglycollate stimulated 11βHSD1^+/+^/apoE^−/−^ and 11βHSD1^−/−^/apoE^−/−^ mice.** **A**) White blood cell (WBC) and **B**) monocyte counts in naïve mice. **C**) White blood cell and **D**) monocyte counts post thioglycollate stimulation. Blood was sampled from 11βHSD1^+/+^/apoE^−/−^ (n = 8) and 11βHSD1^−/−^/apoE^−/−^ (n = 8) mixed sex mice. Values are mean ± SE.
(TIF)

Table S1 **Primer sequences for RT-PCR.**
(TIF)

Table S2 **Differentially expressed genes related to A**) **chemokine signaling, B**) **JAK/STAT signaling, C**) **pattern recognition signaling, D**) **NF-κB signaling, E**) **glucocorticoid signaling from peritoneal macrophages following in vivo thioglycollate challenge in Western diet-fed 11β^+/+^/apoE^−/−^ and 11β^−/−^/apoE^−/−^ mice.**
(TIF)

Table S3 **Differentially expressed genes related to oxidative phosphorylation pathways from peritoneal macrophages following in vivo thioglycollate challenge in Western diet-fed 11β^+/+^/apoE^−/−^and 11β^−/−^/apoE^−/−^ mice.**
(TIF)

## References

[1] Wake DJ, Walker BR (2006) Inhibition of 11beta-hydroxysteroid dehydrogenase type 1 in obesity. Endocrine 29: 101–108. 16622297 10.1385/ENDO:29:1:101

[2] Morgan SA, Tomlinson JW (2010) 11beta-hydroxysteroid dehydrogenase type 1 inhibitors for the treatment of type 2 diabetes. Expert Opin Investig Drugs 19: 1067–1076. 10.1517/13543784.2010.504713 20707593

[3] Hadoke PW, Iqbal J, Walker BR (2009) Therapeutic manipulation of glucocorticoid metabolism in cardiovascular disease. Br J Pharmacol 156: 689–712. 19239478 10.1111/j.1476-5381.2008.00047.x PMC2697748

[4] Hadoke PW, Christy C, Kotelevtsev YV, Williams BC, Kenyon CJ, et al (2001) Endothelial cell dysfunction in mice after transgenic knockout of type 2, but not type 1, 11beta-hydroxysteroid dehydrogenase. Circulation 104: 2832–2837. 11733403 10.1161/hc4801.100077

[5] Preusch MR, Rattazzi M, Albrecht C, Merle U, Tuckermann J, et al (2008) Critical role of macrophages in glucocorticoid driven vascular calcification in a mouse-model of atherosclerosis. Arterioscler Thromb Vasc Biol 28: 2158–2164. 18787189 10.1161/ATVBAHA.108.174128

[6] Fantidis P (2010) The role of the stress-related anti-inflammatory hormones ACTH and cortisol in atherosclerosis. Curr Vasc Pharmacol 8: 517–525. 19485904 10.2174/157016110791330889

[7] Wamil M, Seckl JR (2007) Inhibition of 11beta-hydroxysteroid dehydrogenase type 1 as a promising therapeutic target. Drug Discov Today 12: 504–520. 17631244 10.1016/j.drudis.2007.06.001

[8] Hult M, Elleby B, Shafqat N, Svensson S, Rane A, et al (2004) Human and rodent type 1 11beta-hydroxysteroid dehydrogenases are 7beta-hydroxycholesterol dehydrogenases involved in oxysterol metabolism. Cell Mol Life Sci 61: 992–999. 15095019 10.1007/s00018-003-3476-y PMC11138843

[9] Wamil M, Andrew R, Chapman KE, Street J, Morton NM, et al (2008) 7-oxysterols modulate glucocorticoid activity in adipocytes through competition for 11beta-hydroxysteroid dehydrogenase type. Endocrinology 149: 5909–5918. 18755798 10.1210/en.2008-0420

[10] Brown AJ, Leong SL, Dean RT, Jessup W (1997) 7-Hydroperoxycholesterol and its products in oxidized low density lipoprotein and human atherosclerotic plaque. J Lipid Res 38: 1730–1745. 9323583

[11] Brown AJ, Jessup W (1999) Oxysterols and atherosclerosis. Atherosclerosis 142: 1–28. 9920502 10.1016/s0021-9150(98)00196-8

[12] Livingstone DE, Jones GC, Smith K, Jamieson PM, Andrew R, et al (2000) Understanding the role of glucocorticoids in obesity: tissue-specific alterations of corticosterone metabolism in obese Zucker rats. Endocrinology 141: 560–563. 10650936 10.1210/endo.141.2.7297

[13] Masuzaki H, Paterson J, Shinyama H, Morton NM, Mullins JJ, et al (2001) A transgenic model of visceral obesity and the metabolic syndrome. Science 294: 2166–2170. 11739957 10.1126/science.1066285

[14] Morton NM, Paterson JM, Masuzaki H, Holmes MC, Staels B, et al (2004) Novel adipose tissue-mediated resistance to diet-induced visceral obesity in 11 beta-hydroxysteroid dehydrogenase type 1-deficient mice. Diabetes 53: 931–938. 15047607 10.2337/diabetes.53.4.931

[15] Kotelevtsev Y, Holmes MC, Burchell A, Houston PM, Schmoll D, et al (1997) 11beta-hydroxysteroid dehydrogenase type 1 knockout mice show attenuated glucocorticoid-inducible responses and resist hyperglycemia on obesity or stress. Proc Natl Acad Sci U S A 94: 14924–14929. 9405715 10.1073/pnas.94.26.14924 PMC25139

[16] Morton NM, Holmes MC, Fievet C, Staels B, Tailleux A, et al (2001) Improved lipid and lipoprotein profile, hepatic insulin sensitivity, and glucose tolerance in 11beta-hydroxysteroid dehydrogenase type 1 null mice. J Biol Chem 276: 41293–41300. 11546766 10.1074/jbc.M103676200

[17] Hermanowski-Vosatka A, Balkovec JM, Cheng K, Chen HY, Hernandez M, et al (2005) 11beta-HSD1 inhibition ameliorates metabolic syndrome and prevents progression of atherosclerosis in mice. J Exp Med 202: 517–527. 16103409 10.1084/jem.20050119 PMC2212859

[18] Nuotio-Antar AM, Hachey DL, Hasty AH (2007) Carbenoxolone treatment attenuates symptoms of metabolic syndrome and atherogenesis in obese, hyperlipidemic mice. Am J Physiol Endocrinol Metab 293: E1517–1528. 17878220 10.1152/ajpendo.00522.2007

[19] Paigen B, Morrow A, Holmes PA, Mitchell D, Williams RA (1987) Quantitative assessment of atherosclerotic lesions in mice. Atherosclerosis 68: 231–240. 3426656 10.1016/0021-9150(87)90202-4

[20] Feng B, Zhang D, Kuriakose G, Devlin CM, Kockx M, et al (2003) Niemann-Pick C heterozygosity confers resistance to lesional necrosis and macrophage apoptosis in murine atherosclerosis. Proc Natl Acad Sci U S A 100: 10423–10428. 12923293 10.1073/pnas.1732494100 PMC193577

[21] Howell KW, Meng X, Fullerton DA, Jin C, Reece TB, et al (2011) Toll-like receptor 4 mediates oxidized LDL-induced macrophage differentiation to foam cells. J Surg Res 171: e27–31. 21920554 10.1016/j.jss.2011.06.033

[22] Schmittgen TD, Livak KJ (2008) Analyzing real-time PCR data by the comparative C(T) method. Nat Protoc 3: 1101–1108. 18546601 10.1038/nprot.2008.73

[23] Jawien J, Nastalek P, Korbut R (2004) Mouse models of experimental atherosclerosis. J Physiol Pharmacol 55: 503–517. 15381823

[24] Becker L, Gharib SA, Irwin AD, Wijsman E, Vaisar T, et al (2010) A macrophage sterol-responsive network linked to atherogenesis. Cell Metabolism 11: 125–135. 20142100 10.1016/j.cmet.2010.01.003 PMC2893224

[25] Li AC, Binder CJ, Gutierrez A, Brown KK, Plotkin CR, et al (2004) Differential inhibition of macrophage foam-cell formation and atherosclerosis in mice by PPARalpha, beta/delta, and gamma. J Clin Invest 114: 1564–1576. 15578089 10.1172/JCI18730 PMC529277

[26] Gelissen IC, Brown AJ, Mander EL, Kritharides L, Dean RT, et al (1996) Sterol efflux is impaired from macrophage foam cells selectively enriched with 7-ketocholesterol. J Biol Chem 271: 17852–17860. 8663356 10.1074/jbc.271.30.17852

[27] Tsukamoto K, Kinoshita M, Kojima K, Mikuni Y, Kudo M, et al (2002) Synergically increased expression of CD36, CLA-1 and CD68, but not of SR-A and LOX-1, with the progression to foam cells from macrophages. J Atheroscler Thromb 9: 57–64. 12238639 10.5551/jat.9.57

[28] Gilmour JS, Coutinho AE, Cailhier JF, Man TY, Clay M, et al (2006) Local amplification of glucocorticoids by 11 beta-hydroxysteroid dehydrogenase type 1 promotes macrophage phagocytosis of apoptotic leukocytes. J Immunol 176: 7605–7611. 16751407 10.4049/jimmunol.176.12.7605

[29] Thieringer R, Le Grand CB, Carbin L, Cai TQ, Wong B, et al (2001) 11 Beta-hydroxysteroid dehydrogenase type 1 is induced in human monocytes upon differentiation to macrophages. J Immunol 167: 30–35. 11418628 10.4049/jimmunol.167.1.30

[30] Xu XH, Shah PK, Faure E, Equils O, Thomas L, et al (2001) Toll-like receptor-4 is expressed by macrophages in murine and human lipid-rich atherosclerotic plaques and upregulated by oxidized LDL. Circulation 104: 3103–3108. 11748108 10.1161/hc5001.100631

[31] Edfeldt K, Swedenborg J, Hansson GK, Yan ZQ (2002) Expression of toll-like receptors in human atherosclerotic lesions: a possible pathway for plaque activation. Circulation 105: 1158–1161. 11889007

[32] Choi SH, Harkewicz R, Lee JH, Boullier A, Almazan F, et al (2009) Lipoprotein accumulation in macrophages via toll-like receptor-4-dependent fluid phase uptake. Circ Res 104: 1355–1363. 19461045 10.1161/CIRCRESAHA.108.192880 PMC2741301

[33] Miller YI, Choi SH, Fang L, Harkewicz R (2009) Toll-like receptor-4 and lipoprotein accumulation in macrophages. Trends Cardiovasc Med 19: 227–232. 20382346 10.1016/j.tcm.2010.02.001 PMC2854673

[34] Higashimori M, Tatro JB, Moore KJ, Mendelsohn ME, Galper JB, et al (2011) Role of toll-like receptor 4 in intimal foam cell accumulation in apolipoprotein E-deficient mice. Arterioscler Thromb Vasc Biol 31: 50–57. 20966403 10.1161/ATVBAHA.110.210971 PMC3034636

[35] Du Q, Min S, Chen LY, Ma YD, Guo XL, et al (2012) Major stress hormones suppress the response of macrophages through down-regulation of TLR2 and TLR4. J Surg Res 173: 354–361. 21109260 10.1016/j.jss.2010.10.016

[36] Rickard AJ, Young MJ (2009) Corticosteroid receptors, macrophages and cardiovascular disease. J Mol Endocrinol 42: 449–459. 19158233 10.1677/JME-08-0144

[37] Jaffe IZ, Newfell BG, Aronovitz M, Mohammad NN, McGraw AP, et al (2010) Placental growth factor mediates aldosterone-dependent vascular injury in mice. J Clin Invest 120: 3891–3900. 20921624 10.1172/JCI40205 PMC2964966

[38] Deuchar GA, McLean D, Hadoke PW, Brownstein DG, Webb DJ, et al (2011) 11beta-hydroxysteroid dehydrogenase type 2 deficiency accelerates atherogenesis and causes proinflammatory changes in the endothelium in apoe−/− mice. Endocrinology 152: 236–246. 21106873 10.1210/en.2010-0925 PMC3977042

[39] Vats D, Mukundan L, Odegaard JI, Zhang L, Smith KL, et al (2006) Oxidative metabolism and PGC-1beta attenuate macrophage-mediated inflammation. Cell Metab 4: 13–24. 16814729 10.1016/j.cmet.2006.05.011 PMC1904486

[40] Garedew A, Moncada S (2008) Mitochondrial dysfunction and HIF1alpha stabilization in inflammation. J Cell Sci 121: 3468–3475. 18827009 10.1242/jcs.034660

[41] Garedew A, Henderson SO, Moncada S (2010) Activated macrophages utilize glycolytic ATP to maintain mitochondrial membrane potential and prevent apoptotic cell death. Cell Death Differ 17: 1540–1550. 20339378 10.1038/cdd.2010.27

[42] Yao PM, Tabas I (2000) Free cholesterol loading of macrophages induces apoptosis involving the fas pathway. J Biol Chem 275: 23807–23813. 10791964 10.1074/jbc.M002087200

[43] Salvayre R, Auge N, Benoist H, Negre-Salvayre A (2002) Oxidized low-density lipoprotein-induced apoptosis. Biochim Biophys Acta 1585: 213–221. 12531556 10.1016/s1388-1981(02)00343-8

[44] Manning-Tobin JJ, Moore KJ, Seimon TA, Bell SA, Sharuk M, et al (2009) Loss of SR-A and CD36 activity reduces atherosclerotic lesion complexity without abrogating foam cell formation in hyperlipidemic mice. Arterioscler Thromb Vasc Biol 29: 19–26. 18948635 10.1161/ATVBAHA.108.176644 PMC2666043

[45] Tabas I (2010) Macrophage death and defective inflammation resolution in atherosclerosis. Nat Rev Immunol 10: 36–46. 19960040 10.1038/nri2675 PMC2854623

[46] Seimon T, Tabas I (2009) Mechanisms and consequences of macrophage apoptosis in atherosclerosis. J Lipid Res 50 Suppl: S382–387 10.1194/jlr.R800032-JLR200 PMC2674693 18953058

[47] Thorp E, Tabas I (2009) Mechanisms and consequences of efferocytosis in advanced atherosclerosis. J Leukoc Biol 86: 1089–1095. 19414539 10.1189/jlb.0209115 PMC2774877

[48] Tahrani AA, Bailey CJ, Del Prato S, Barnett AH (2011) Management of type 2 diabetes: new and future developments in treatment. Lancet 378: 182–197. 21705062 10.1016/S0140-6736(11)60207-9

