# Supplementary figures and images for: 11β-Hydroxysteroid Dehydrogenase Type 1 Gene Knockout Attenuates Atherosclerosis and In Vivo Foam Cell Formation in Hyperlipidemic apoE−/− Mice
Source: PLoS One. 2013 Feb 1;8(2):e53192. doi: 10.1371/journal.pone.0053192 (PMC3562192; doi:10.1371/journal.pone.0053192)

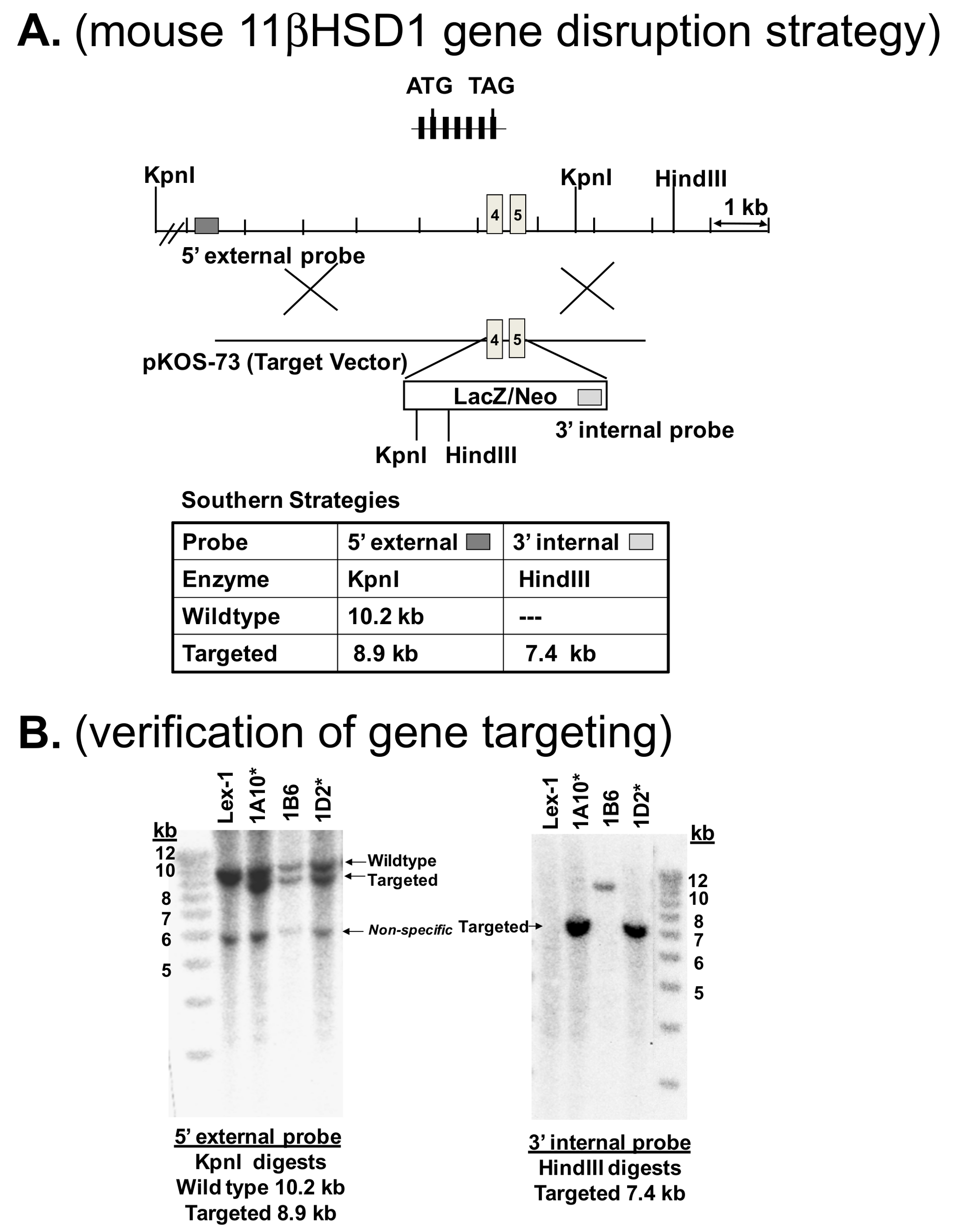

Supplement: Figure S1 — Targeted disruption of the 11βHSD1 gene locus. A) Targeting strategy used to disrupt the 11βHSD1 locus. Homologous recombination (represented by X) between the targeting vector and the 11βHSD1 gene results in the replacement of exons 4 and 5 with the selection cassette. The 11βHSD1 targeting vector was derived using the Lambda KOS system. The Lambda KOS phage library, arrayed into 96 superpools, was screened by PCR using exons 4 and 5-specific primers Hsd-1 (5′-AGGTAGTGTCTCGCTGCCTT-3′) and Hsd-3 (5′-CTTCGCACAGAGTGGATGTC-3′). PCR-positive phage superpools were plated and screened by filter hybridization using the 306 bp amplicon derived from primers Hsd-1 and Hsd-3 as a probe. Three pKOS genomic clones, pKOS-63, pKOS-73 and pKOS-82, were isolated from the library screen and confirmed by sequence and restriction analysis. The yeast selection cassette and pKOS-73 were co-transformed into yeast. Clones that had undergone homologous recombination to replace a 408 bp region containing exon 4 and exon 5 with the yeast selection cassette were isolated. The yeast cassette was subsequently replaced with a LacZ/Neo selection cassette to complete the 11βHSD1 targeting vector. The Not I linearized targeting vector was electroporated into 129/SvEvBrd (Lex-1) ES cells. G418/FIAU resistant ES cell clones were isolated, and correctly targeted clones were identified and confirmed by Southern analysis using a 328 bp 5′ external probe (9/10), generated by PCR using primers Hsd-9 (5′-CAATGCATCCATGCGCCTGAA-3′) and Hsd-10 (5′-AGAGACCAGACATTAGGACAC-3′) and a 607 bp 3′ internal probe (Neo5/2), amplified by PCR using primers Neo-5 (5′-GGCAGCGCGGCTATCGTG-3′) and Neo-2 (5′-TCAGAAGAACTCGTCAAG-3′). B) Southern hybridization indicating proper gene targeting in the embryonic stem cell clones. Clones 1A10 and 1D2 were selected for blastocyst injections, as denoted by the asterisk symbols. Lex-1 represents untransfected embryonic stem cell DNA. (TIF) [file pone.0053192.s001.tif]

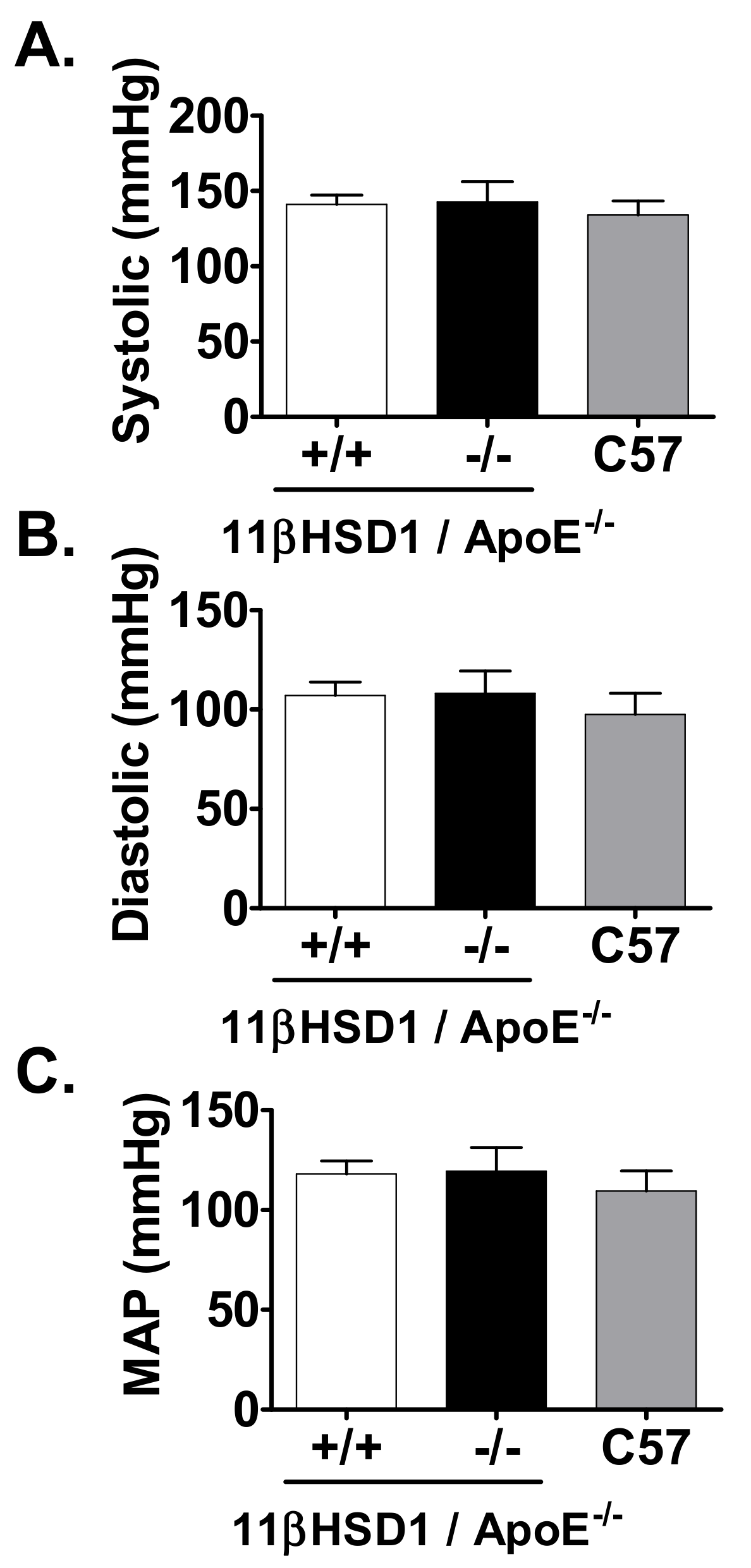

Supplement: Figure S4 — Blood pressures in hyperlipidemic 11βHSD1+/+/apoE−/− and 11βHSD1−/−/apoE−/− mice. A) Systolic, B) diastolic and C) mean arterial pressure (MAP) in mixed sex 11βHSD1+/+/apoE−/− (n = 8) and 11βHSD1−/−/apoE−/− (n = 7) mice. Blood pressures were measured in conscious mice using a non-invasive computerized tail cuff system (CODA Non-Invasive Blood Pressure Monitor, Kent Scientific Corporation, Torrington, CT). Mice were conditioned to tail cuff instrumentation over several days to control for stress. As a normolipidemic reference, blood pressures were also measured in chow-fed male C57BL/6 mice (n = 7; noted as “C57” in bar graph). Data for individual animals represent the average of at least 5 acquisitions. Values are mean ± SE. (TIF) [file pone.0053192.s004.tif]

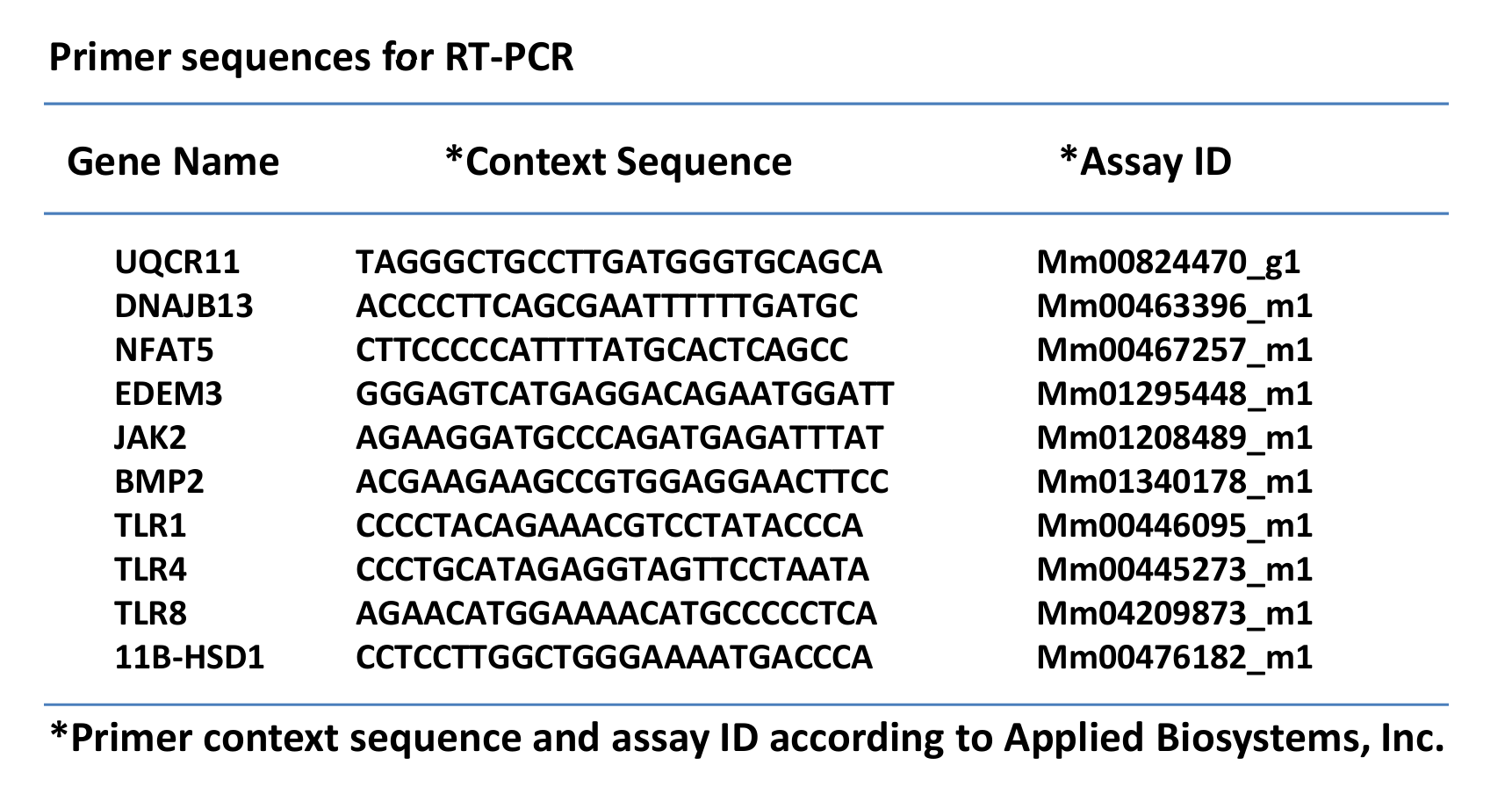

Supplement: Table S1 — Primer sequences for RT-PCR. (TIF) [file pone.0053192.s008.tif]

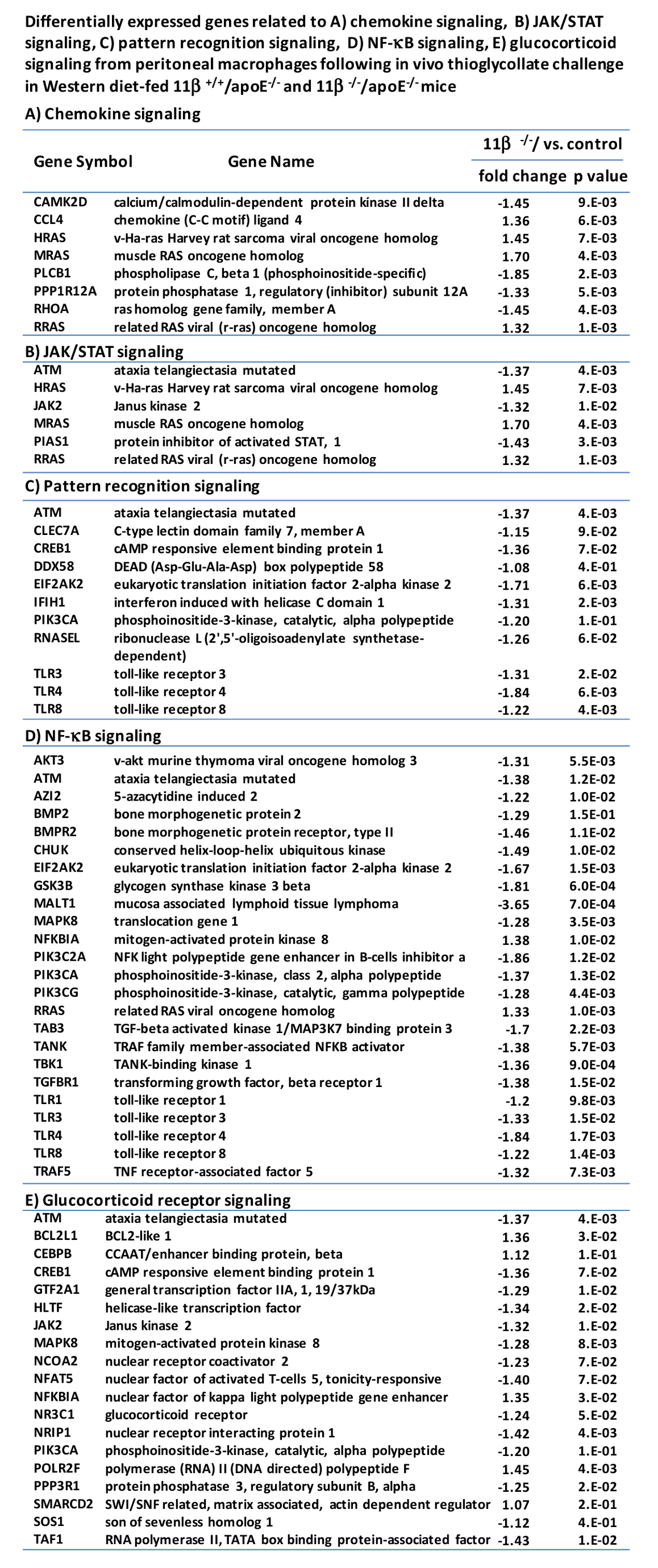

Supplement: Table S2 — Differentially expressed genes related to A) chemokine signaling, B) JAK/STAT signaling, C) pattern recognition signaling, D) NF-κB signaling, E) glucocorticoid signaling from peritoneal macrophages following in vivo thioglycollate challenge in Western diet-fed 11β+/+/apoE−/− and 11β−/−/apoE−/− mice. (TIF) [file pone.0053192.s009.tif]

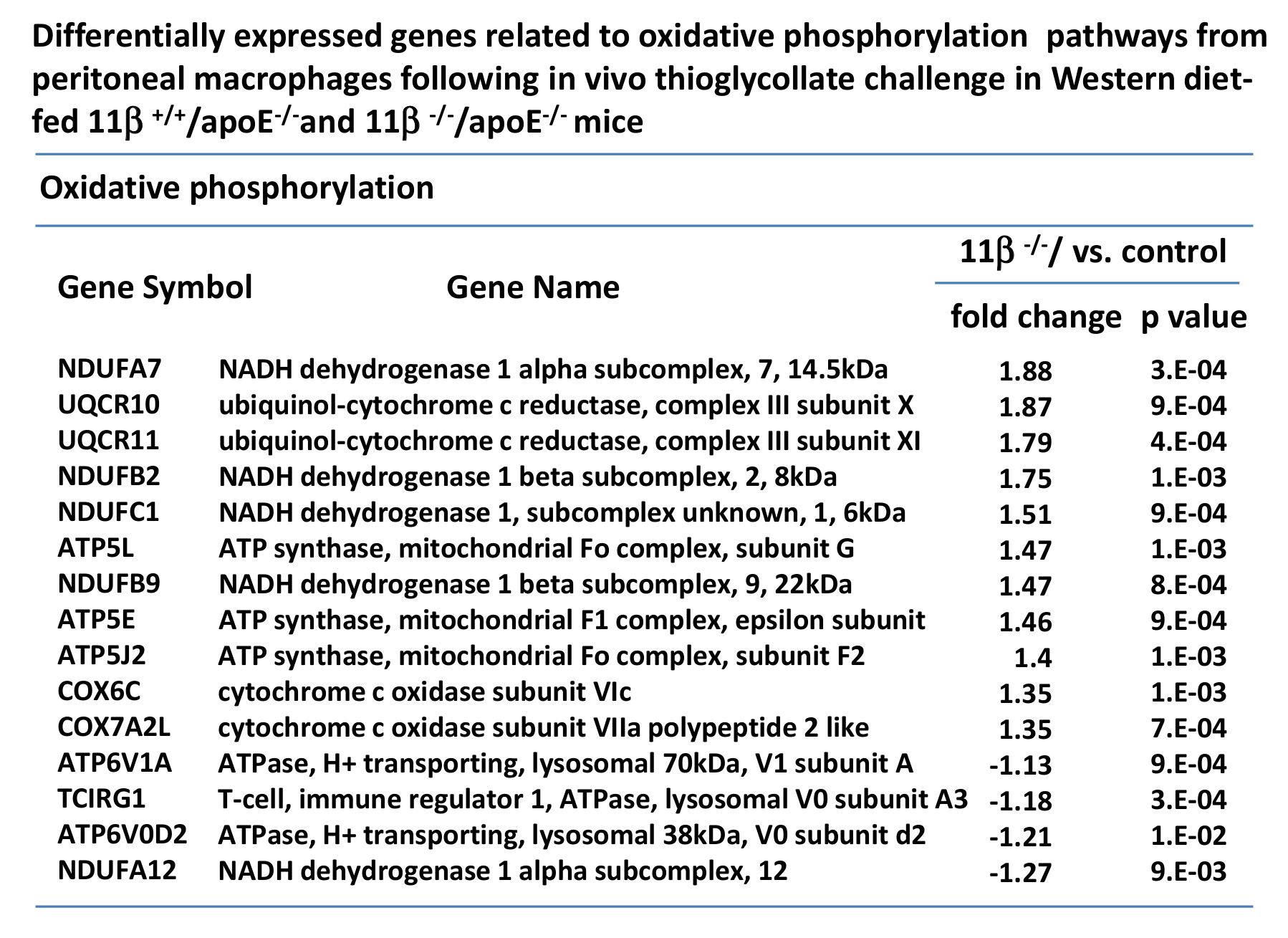

Supplement: Table S3 — Differentially expressed genes related to oxidative phosphorylation pathways from peritoneal macrophages following in vivo thioglycollate challenge in Western diet-fed 11β+/+/apoE−/−and 11β−/−/apoE−/− mice. (TIF) [file pone.0053192.s010.tif]
